# Comparison of survival analysis approaches to modelling age at first sex among youth in Kisesa Tanzania

**DOI:** 10.1371/journal.pone.0289942

**Published:** 2023-09-07

**Authors:** Jacqueline Materu, Eveline T. Konje, Mark Urassa, Milly Marston, Ties Boerma, Jim Todd

**Affiliations:** 1 Program of Sexual and Reproductive Health, National Institute for Medical Research, Mwanza Centre, Mwanza, Tanzania; 2 Department of Biostatistics, Epidemiology and Behavioral Sciences, School of Public Health, Catholic University of Health, and Allied Sciences, Mwanza, Tanzania; 3 Department of Population Health, London School of Hygiene and Tropical Medicine, London, United Kingdom; 4 Institute for Global Public Health, University of Manitoba, Manitoba, Canada; University of Salamanca, SPAIN

## Abstract

**Background:**

Many studies analyze sexual and reproductive event data using descriptive life tables. Survival analysis has better power to estimate factors associated with age at first sex (AFS), but proportional hazards models may not be right model to use. This study used accelerated failure time (AFT) models, restricted Mean Survival time model (RMST) models, with semi and non-parametric methods to assess age at first sex (AFS), factors associated with AFS, and verify underlying assumptions for each analysis.

**Methods:**

Self-reported sexual debut data was used from respondents 15–24 years in eight cross-sectional surveys between 1994–2016, and from adolescents’ survey in an observational community study (2019–2020) in northwest Tanzania. Median AFS was estimated in each survey using non-parametric and parametric models. Cox regression, AFT parametric models (exponential, gamma, generalized gamma, Gompertz, Weibull, log-normal and log-logistic), and RMST were used to estimate and identify factors associated with AFS. The models were compared using Akaike information criterion (AIC) and Bayesian information criterion (BIC), where lower values represent a better model fit.

**Results:**

The results showed that in every survey, the Cox regression model had higher AIC and BIC compared to the other models. Overall, AFT had the best fit in every survey round. The estimated median AFS using the parametric and non-parametric methods were close. In the adolescent survey, log-logistic AFT showed that females and those attending secondary and higher education level had a longer time to first sex (Time ratio (TR) = 1.03; 95% CI: 1.01–1.06, TR = 1.05; 95% CI: 1.02–1.08, respectively) compared to males and those who reported not being in school. Cell phone ownership (TR = 0.94, 95% CI: 0.91–0.96), alcohol consumption (TR = 0.88; 95% CI: 0.84–0.93), and employed adolescents (TR = 0.95, 95% CI: 0.92–0.98) shortened time to first sex.

**Conclusion:**

The AFT model is better than Cox PH model in estimating AFS among the young population.

## Introduction

Age at first sex (AFS) is a critical indicator for measuring the onset of an adolescent’s sexual and reproductive life. The onset of sex is a normative step in adolescent sexual development [1]. However, early sex is associated with negative outcomes, including unwanted pregnancies and sexually transmitted infections (STIs) [1–3]. Once young people become sexually active, they are at greater risk of having multiple, usually consecutive, short-term sexual relationships, and inconsistent use of condoms, putting them at higher risk of contracting HIV and other STIs [4, 5]. Accurate monitoring and estimation of AFS has become increasingly important in measuring behavioral changes in HIV prevention and family planning programs [5].

Data on AFS are often collected through self-reports in nationally representative household surveys to track health and population indicators such as the Demographic and Health Surveys (DHS) [6, 7]. Many challenges have been identified in the measurement and modelling of AFS, as studies have shown inconsistent trends that were difficult to interpret [8, 9]. Measurement challenges encompass recall biases, social desirability responses, and a lack of accurate information [10, 11]. Modelling challenges involve failing to account for age censoring [12–14], and the use of inappropriate analysis methods such as logistic regression when dealing with AFS outcome.

Some studies in the existing literature [8, 9] have provided arguments highlighting the distinct advantages of survival analysis in assessing the initiation of sexual and reproductive events because of the distinctive characteristics of the data and its population. AFS is most often estimated using time to event methods, and frequently, a Cox’s proportional hazards (PH) model is applied to estimate factors associated with AFS [8, 9, 15–18]. The Cox PH model necessitates the fulfillment of the assumption of hazard function proportionality. In the case of AFS, the Cox PH assumption is improbable to be satisfied since all individuals will eventually initiate sex, making it impossible for one group to consistently possess a greater risk (or hazard) than another group. When the Cox PH assumption is violated, the utilization of the standard Cox PH model becomes inappropriate, as it can introduce significant bias and result in diminished statistical power when estimating or inferring the impact of a specific risk factor on desired outcomes [19]. According to a review of survival analysis in cancer journals, it was reported that only 5% of all studies using the Cox PH model examined the underlying PH assumption [20]. Similarly, some studies have used Cox’s PH model to find factors associated with AFS without clearly stating whether they examined the PH assumption and if the assumption was met or not [8, 9, 15–18]. While time-to-event methods have been optimal for modeling and estimating factors related to AFS, other studies have classified AFS into predefined time intervals and used standard logistic regression for analysis to get the odds of a person having sex before a certain age [21]. Although logistic regression in analysing AFS might generate results, the results will be biased because important information would be left out due to censoring. Logistic regression does not consider censoring observations (e.g., excluding never had sex in the analysis), it treated as a missing and leads to losing percentage of data, power and biased estimates [22]. Others used life table methods and Kaplan Meier methods, which both considered censoring. The life table method provides a direct and easy method of analysing AFS at pre-determined intervals, and it takes into account censored observations [23, 24]. However, the intervals in the life table are based on calendar time (fixed-length interval) instead of observed events. Since this method is based on these calendar intervals and not based on individual events or censoring times, it uses the average risk set size per interval to estimate survival and must assume that censoring occurred uniformly over the calendar time interval [25]. Due to this reason, the life table method sometimes is not as precise as the Kaplan-Meier method, but the results will be similar in very large samples. Accelerated Failure Time (AFT) analyses the time to event directly, rather than the survival function used in PH models, with AFT assuming a multiplicative effect of covariates on survival time rather than survival hazard [26]. Despite being less utilized compared to Cox PH in the analysis of AFS, the AFT model is considered a highly adaptable survival model for investigating the AFS. This model provides versatility in its assumptions and parameterization, making it a robust tool for studying AFS outcomes. It can accommodate various distributions and shapes of hazards (e.g., increasing, decreasing, or constant), enabling estimation of the effects of covariates, handling time-varying covariates, and facilitating prediction and inference [27]. Restricted Mean Survival Time (RMST) is the average event-free time over a fixed follow-up period and can be interpreted as the average event-free survival time to a predetermined key time point [28, 29]. It corresponds to the area under the Kaplan-Meier curve from the beginning of the study through that time point. Whether or not the PH assumption is violated, the RMST difference is valid and interpretable.

Assessing age at first sexual intercourse and trends in AFS over time requires the use of the most appropriate statistical methods [8, 9]. This study aimed to analyse age at first sexual intercourse from eight-surveys during 1994–2016 among youth 15–24 years and an adolescent survey (2019–2020) among adolescents 15–19 years in Northwest Tanzania. Different statistical methods, including life table, Kaplan-Meier, Cox PH, and AFT, were used to estimate the median time of AFS and investigate their effects on AFS trends. Also, the estimated coefficient factors associated with AFS were compared using Cox PH, RMST, and AFT. The performance of Cox PH and AFT in modeling factors related to AFS was evaluated, along with an assessment of whether the underlying assumptions behind the models were met.

## Methods

### Data source

We used data from eight surveys in Kisesa observational community study (KOCS) conducted between 1994–2016 and adolescent survey conducted in 2019–2020. KOCS is located 20 kilometers east of Mwanza city in Magu district and nested within a Health and Demographic Surveillance System (HDSS). Sampling strategies and survey methods for KOCS have been described in detail elsewhere [11]. Briefly, the surveys included all adults aged 15 years and above who are listed in the respective HDSS follow-up rounds, with data collected using a structured questionnaire. Our analysis focused on adolescents and young adults aged 15 to 24 years, who attended at least one survey from 1994 to 2016. These surveys were cross-sectional for each round, and each round was analysed separately. Additional data were used with the final best-fit model at the end of eight rounds of analysis to observe factors associated with AFS.

**Fig 1:**
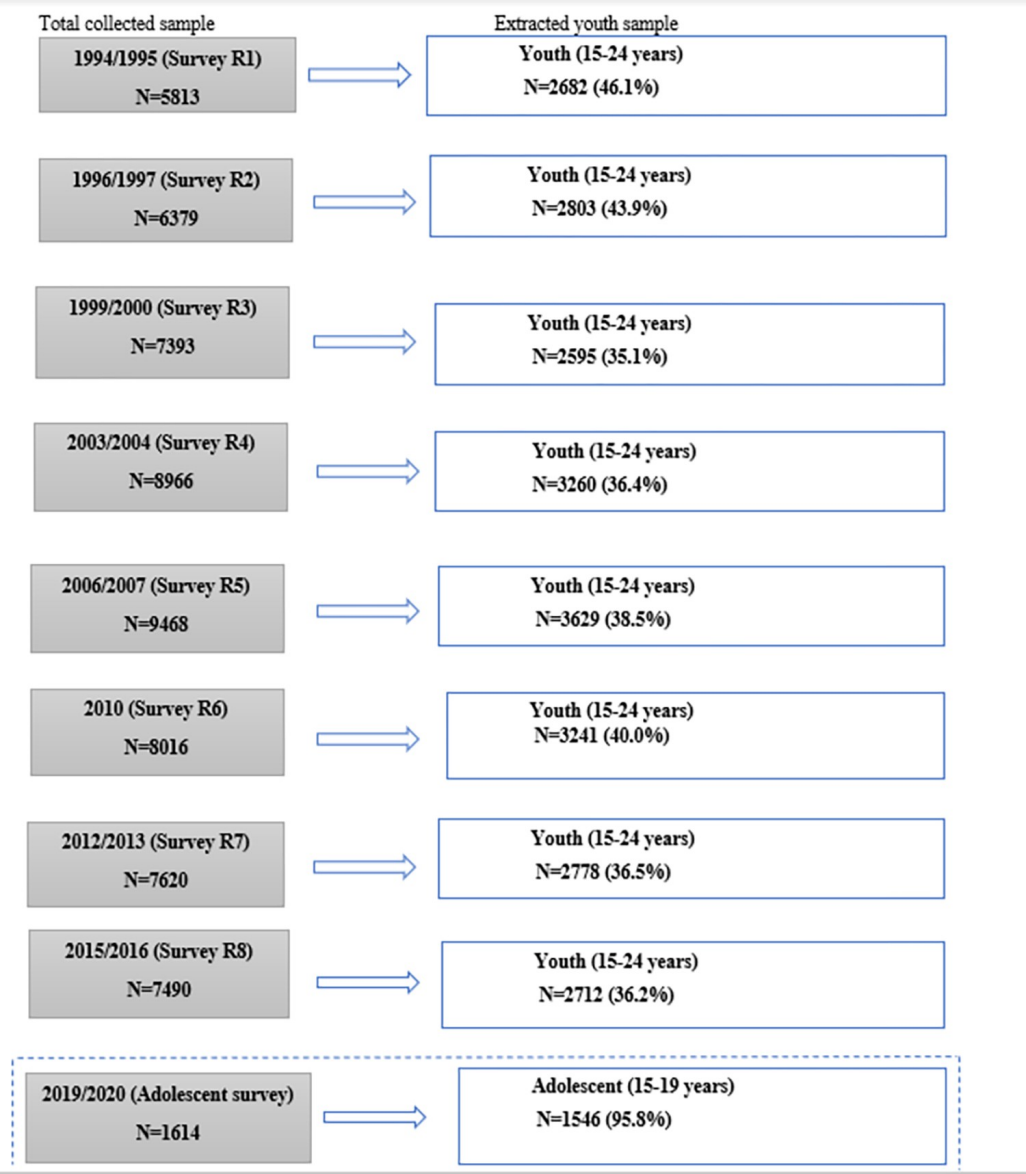
Sample collected and extracted in each survey (1994–2016) and adolescent survey (2019/2020).

The additional data comes from a survey of adolescents (15–19 years) conducted in the same area between September 2019 and January 2020, using Audio Computer-Assisted Self Interviewing (ACASI) as a data collection tool. The ACASI method was used as the survey tool intended to collect sensitive data on reproductive health and risk behaviors of youth in Kisesa. The sample size per survey round ranged from 5813 to 8966, of which 2592 to 3287 were youth participants (15–24 years) while the adolescent survey included 1546 adolescents aged 15–19 (Fig 1).

### Data management

From each survey round, participants aged 15–24 at the time of the survey were included in this study. The individual-level data were harmonised to a common data specification over all eight rounds. Where there were inconsistencies between survey rounds in question response categories or wording, we coded a variable that was comparable to the data available in each round (Table 1). Where differences in questions between the survey rounds were too large to generate a comparable variable, we excluded that variable from the analysis for that particular round. For adolescent survey data, all participants aged 15–19 years were included in the analysis.

**Table 1:** Coding and explanation of independent variables.

Variable	Description	Categories
	**Categorization of survey round 1 to 8**	
Sex	Sex of the participant	0 = Male; 1 = Female
Age	Age of the participants in years	0 = 15–19 years; 1 = 20–24 years
Residence	Participant’s residence area	0 = Rural [Igekemaja or Kitumba or Isangijo or Ihayabuyaga or Welamasonga]; 1 = Semi-urban/urban [Kanyama or Kisesa]
Formal education	If participants have formal education	0 = No/ 0 years of education; 1 = Yes/ 1+years of education
Education level	Participants completed years of education	0 = No education (0 years);1 = Primary [1-4/5-7 years]; 2 = Secondary/higher/other [8+years]
Religion	Participant’s religion	0 = Traditional/non-religious/other; 1 = Muslim; 2 = Christian [Catholic/protestant/Evangelical]
Marital status	Participant’s current marital status	0 = Never married or been in cohabiting union, 1 = Monogamously married or cohabiting/Polygamously married or cohabiting, 2 = widowed/separated/divorced
Employment status	If participant perform any work that helps him/her or household earn money	0 = Unemployed [No, I just look after the house/ No, I am too ill to work/No, I am too old to work/No other reason]; 1 = Student [No, I am still a student]; 2 = Employed [Yes]
	**Categorization of Adolescent survey**	
Current in school	Are you in school this year?	0 = No, 1 = Yes
Current level of education	What level of education are you currently studying	0 = Not in school, 1 = Primary school, 2 = Secondary and above
Employment status	Are you currently employed or do you have a job that pays you any salary or wages?	0 = No, 1 = Yes
Religion	Participant’s denomination	0 = other/not belong to any, 1 = Muslim, 2 = Christian [Catholic/AIC/EAGT/Sabbath/Lutheran]
How important is religion	How important is religion to you?	0 = Not important/I don’t know it’s importance, 1 = Important, 2 = Very important
Live with mother	Do you live with your mother?	0 = No, 1 = Yes
Live with father	Do you live with your father?	0 = No, 1 = Yes
Own mobile	Do you own mobile phone?	0 = No, 1 = Yes
Ever use alcohol	Have you ever used alcohol?	0 = No, 1 = Yes

### Measures

The Kisesa survey (in all rounds except round three, where age at first sex question was not asked) asked two questions to determine the end event (age at first sex, or censoring): “Have you ever had sex? (i.e., ever had sexual intercourse)” and “How old were you when you first had sexual intercourse?”. Similar questions were asked in the adolescent survey. The dependent variable of this analysis was AFS, which is the age in years from birth to the event. The end event was AFS, defined as age at first sexual intercourse among those who ever had sex (retrospective reports of AFS). Those who reported not yet sexually active were right censored at their age at interview and for those who had ever had sex, the failure event occurred at the reported age at first sex.

The main independent variables were the place of residence, sex, level of education, religion, age, employment status and marital status. In all surveys, structured questions were used, with the full questionnaires from the surveys available by request, and the selected questions from the eight surveys and from the adolescent survey are available in S1 Questionnaire. The categorization of selected independent variables for each round and for the adolescent survey was presented in Table 1.

### Statistical analysis

From each of the eight surveys, the frequencies and percentages of the demographic characteristics for the youth were presented similarly for the adolescent surveys. The median age at first sex was estimated separately for males and females using both non-parametric (the life table method and Kaplan-Meier estimator (KM)) and parametric methods (final selected model). For groups in which less than 50% had intercourse, the median survival time could only be estimated using parametric methods. The proportional hazard assumption was checked using Schoenfeld residuals (PH test) and assumption would be met if the p-value is greater than 0.05. The homogeneity of the censorship mechanism was assessed using Kaplan-Meier curves and a log-rank test. The curves showing significant separation or a small p-value from the log-rank test supported the violation of the homogeneity of the censorship mechanism, suggesting that censorship patterns differed between groups.

The modelling used data from both sexes together, for separate models for each survey round except for survey round 3, which could not modelled because the important key questions defining the outcome of interest were not asked in that round.

Univariable and multivariable analysis using Cox PH regression was fitted to investigate the relation between covariates and time of first sex. The Cox PH is a semi parametric survival model where the baseline takes no distribution [30]. It is widely used and preferable to fully parametric models, as it contains both parametric and non-parametric parts with broad flexibility. The hazard rate in a Cox PH model is defined as:

$$\frac{\lambda (t|x,\beta )}{\lambda_{0}(t)}=exp(x^{T}\beta )$$

*Where λ(t|x*,*β) represents the hazard rate at time t for an individual with covariate values x and coefficient vector β*, *λ*_*0*_*(t) is the baseline hazard (hazard value when the value of all covariates is zero)*, *x*^*T*^*β is the dot product of the covariate vector of independent variables (sex*, *education*, *place of residence*. *etc*.*) and β is the vector of coefficients*.

We also use restricted mean survival time (RMST). RMST measure the average event-free survival time in a pre-specified time, defined as:

$$RMST(t^{*})=E(x)=E[min(T,t^{*})]=\int_{0}^{t^{*}}S(t)dt$$

Where T is a non-negative random variable that represents the failure time from homogenous population, t* is a pre-specified time point of interest (23 years was used), x is the minimum of T and t* and S(t) is the survival function. Then the RMST is defined as the expected value of x, which can be evaluated by the area under the survivor function over [0, t*]

Log-linear of RMST was fitted using pseudo-value method looking for an underlying relation between the RMST and independent variables.


$$log[RMST(t^{*})]=x^{\prime }\beta$$

Where, x is the vector of independent variables (sex, education, place of residence. etc.) and β is the vector of coefficients.

The pseudo values (PV) method uses jack-knife leave -one-out estimation to generate pseudo values, which are then used to model the effects of covariates on the outcome of interest through a generalized estimating equation. The PV method is not constrained by the assumption of homogeneity of the censorship mechanism [31, 32].

Additionally, the accelerated failure time (AFT) model was fitted. This model explains a linear relationship between the logarithm of the survival time and the covariates, defined as:

$$\log\left[\left(T\right)\right]=\beta_{0}+x^{\prime }\beta +\varepsilon$$

*Where*, *T is a survival time*, *x is the vector of independent variables (sex*, *education*, *place of residence*. *etc*.*)*, *β*_*0*_ *is the intercept*, *β is the vector of coefficients and ε is a random error term assumed to follow some parametric distribution*.

We fitted seven types of AFT models (i.e., Weibull, exponential, gamma, generalized gamma, Gompertz, log-normal and log-logistic) which assume different distributions for the random error term in the model. Although parametric models are very well suited for analyzing survival data, there are relatively few probability distributions for survival time that can be used with these models. The choice of which distribution to consider is determined by prior assumptions or scientific knowledge about the data. In this analysis, we use Weibull (a very flexible distribution and its hazard rate can be monotonically increasing or decreasing or constant), log-normal (hazard increases and later decreases), log-logistic (hazard rate first increases and then decreases and can sometimes be hump-shaped), exponential (assuming a constant hazard) and others. The log-logistic distribution is very similar in shape to the log-normal distribution, but it has the advantage of having simple algebraic expressions for its survival and hazard functions and a closed form for its distribution function. The results of the models were observed and compared. The performance of the Cox PH and AFT models was compared using the Akaike information criterion (AIC) and the Bayesian information criterion (BIC). The model with the smallest AIC or BIC was considered the most appropriate. However, the comparison of RMST with Cox PH and AFT using AIC/BIC was not conducted since RMST uses an estimating equation as its estimation method, rather than likelihood or partial likelihood. However, the estimated coefficients and final inference related to factors associated with AFS were compared with other models to assess the similarity of the results with Cox PH and AFT models. This comparison helped highlight the potential usefulness of RMST as an alternative model in situations where Cox PH and AFT models are not feasible for this type of data. All analyses were performed using R version 4.2.1 with a significance level of P<0.05.

### Ethics statement

The current study received ethical approval from the Catholic University Health and Allied Sciences (CUHAS) and the Bugando Medical Centre (BMC) Research Ethics and Review Committee (CREC/585/2022). This study involved secondary data analysis from KOCS, which received its ethical approval from the Lake Zone Institutional Review Board (LZIRB), the medical Research Coordinating Committee of Tanzania and the London School of Hygiene and Tropical Medicine. Participants in the KOCS were informed about the study’s objectives, which included the prospective utilization of their data for subsequent research. Prior to conducting the survey interviews, explicit written consent was obtained from the participants. The process involved the oral presentation of the informed consent explanation to all attendees of the surveys, allowing them adequate time to express their agreement or disagreement and affix their signature to the relevant section of the cover sheet. In the case of participants aged 15–17 years, informed consent was presented to their parents or guardians, who were given opportunities to consent or refuse their children’s participation in the surveys. Although the participants in the survey who were minors (aged 15–17), obtained approval from their parents or guardians, they were also required to assent before involving them in the study. No personally identifiable information was used in the survey data, instead unique anonymized identification numbers were used.

## Results

### Demographic characteristics of the participants

Tables 2 and 3 show the demographic characteristics by survey rounds for girls and boys, respectively. The numbers available for some variables are slightly lower than the total number due to missing responses. Over half of the participants resided in rural areas for males and females in each round. The proportion of respondents who were young adults (20–24 years) fell from 54.8% to 38.8% for females between 1996 to 2016 and slightly smaller decrease for males between 1994 to 2000 and 2004 to 2010 (Table 3). For females, the proportion with secondary or higher education increased from 2.3% to 42.8% between 1999 to 2016 and decrease in proportion with no education from 19.3% to 9.0% during the same period (Table 2). Similarly, the proportion of males with secondary or higher education level increased from 2.7% in 1996 to 53.3% in 2016.

**Table 2:** Demographic characteristics of participants for survey rounds 1 to 8 (15–24 years) for girls.

Years	1994/1995	1996/1997	1999/2000	2003/2004	2006/2007	2010	2012/2013	2015/2016
	Survey1	Survey 2	Survey 3	Survey 4	Survey 5	Survey 6	Survey 7	Survey 8
**Total in Girls**	1353	1408	1356	1660	1704	1724	1473	1517
**Age group (years)**								
15–19	665 (49.2)	636 (45.2)	673 (49.6)	857 (51.6)	984 (57.7)	1039 (60.3)	903 (61.3)	928 (61.2)
20–24	688 (50.8)	772 (54.8)	683 (50.4)	803 (48.4)	720 (42.3)	685 (39.7)	570 (38.7)	589 (38.8)
**Residence**								
Rural	857 (63.3)	861 (61.2)	907 (66.9)	892 (53.7)	1063 (62.4)	1144 (66.4)	1029 (69.9)	892 (58.8)
Semi-urban/urban	496 (36.7)	547 (38.8)	448 (33.0)	768 (46.3)	641 (37.6)	580 (33.6)	444 (30.1)	621 (40.9)
**Marital status**								
Never married	682 (50.4)	585 (41.5)	583 (43.0)	718 (43.3)	726 (42.6)	986 (57.2)	891 (60.5)	984 (64.9)
Monogamous/ polygamous	617 (45.6)	732 (52.0)	716 (52.8)	845 (50.9)	375 (22.0)	690 (40.0)	545 (37.0)	488 (32.2)
Widow/Separated	54 (4.0)	91 (6.5)	57 (4.2)	61 (3.7)	47 (2.8)	43 (2.5)	36 (2.4)	45 (3.0)
**Formal education**								
No	165 (12.2)	217 (15.4)	262 (19.3)	267 (16.1)	295 (17.3)	263 (15.3)	158 (10.7)	137 (9.0)
Yes	1188 (87.8)	1191 (84.6)	1094 (80.7)	1393 (83.9)	1408 (82.6)	1460 (84.7)	1315 (89.3)	1380 (91.0)
**Level of formal education**								
No education	165 (12.2)	217 (15.4)	262 (19.3)	267 (16.1)	297 (17.4)	263 (15.3)	158 (10.7)	137 (9.0)
Primary education (1-4/5-7)	1156 (85.4)	1154 (82.0)	1063 (78.4)	1276 (76.9)	1247 (67.3)	1084 (62.9)	898 (61.0)	730 (48.2)
Secondary or higher education	32 (2.4)	37 (2.6)	31 (2.3)	117 (7.0)	260 (15.3)	376 (21.8)	417 (28.3)	650 (42.8)
**Employment status**								
Unemployed	N/A	N/A	N/A	N/A	217 (12.7)	87 (5.0)	258 (17.5)	281 (18.5)
Student					663 (38.9)	710 (41.2)	590 (40.1)	634 (41.8)
Employed					823 (48.3)	919 (53.3)	625 (42.4)	602 (39.7)
**Religion**								
Traditional/other	180 (13.3)	158 (11.2)	112 (8.3)	97(5.8)	70 (4.1)	45 (2.6)	48 (3.3)	66 (4.4)
Muslim	39 (2.9)	36 (2.6)	29 (2.1)	44 (2.7)	34 (2.0)	41 (2.4)	32 (2.2)	44 (2.9)
Christian	1134 (83.8)	1214 (86.2)	1215 (89.6)	1519 (91.5)	1599 (93.8)	1635 (94.8)	1393 (94.6)	1407 (92.7)

**Table 3:** Demographic characteristics of participants for survey rounds 1 to 8 (15–24 years) for boys.

Years	1994/1995	1996/1997	1999/2000	2003/2004	2006/2007	2010	2012/2013	2015/2016
	Survey1	Survey 2	Survey 3	Survey 4	Survey 5	Survey 6	Survey 7	Survey 8
**Total in Boys**	1329	1395	1239	1600	1581	1498	1305	1195
**Age group (years)**								
15–19	759 (57.1)	779 (55.8)	692 (55.9)	931 (58.2)	1066 (67.4)	1042 (69.6)	881 (67.5)	818 (68.5)
20–24	570 (42.9)	616 (44.2)	547 (44.1)	669 (41.8)	515 (32.6)	456 (30.4)	424 (32.5)	377 (31.5)
**Residence**								
Rural	915 (68.8)	894 (64.1)	800 (64.6)	941 (58.8)	924 (58.4)	1019 (68.0)	990 (75.9)	760 (63.6)
Semi-urban/urban	414 (31.2)	501 (35.9)	439 (35.4)	659 (41.2)	657 (41.6)	479 (32.0)	315 (24.1)	433 (36.2)
**Marital status**								
Never married	1172 (88.2)	1208 (86.6)	1060 (85.6)	1396 (87.2)	1208 (76.4)	1391 (92.9)	1218 (93.3)	1108 (92.7)
Monogamous/ polygamous	147 (11.1)	160 (11.5)	167 (13.5)	187 (11.7)	95 (6.0)	89 (5.9)	84 (6.4)	81 (6.8)
Widow/Separated	10 (0.8)	27 (1.9)	12 (1.0)	8 (0.5)	14 (0.9)	4 (0.3)	3 (0.2)	6 (0.5)
**Formal education**								
No	109 (8.2)	125 (9.0)	153 (12.3)	154 (9.6)	167 (10.6)	96 (6.4%)	117 (9.0)	80 (6.7)
Yes	1220 (91.8)	1270 (91.0)	1086 (87.7)	1146 (90.4)	1414 (89.4)	1254 (83.7)	1188 (91.0)	1115 (93.3)
**Level of formal education**								
No education	109 (8.2)	125 (9.0)	153 (12.3)	154 (9.6)	169 (10.7)	96 (6.4)	117 (9.0)	80 (6.7)
Primary education (1-4/5-7)	1170 (88.0)	1233 (88.4)	1043 (84.2)	1290 (80.6)	1059 (67.0)	664 (54.2)	726 (55.6)	478 (40.0)
Secondary or higher education	50 (3.8)	37 (2.7)	43 (3.5)	156 (9.8)	353 (22.3)	590 (39.4)	462 (35.4)	637 (53.3)
**Employment status**								
Unemployed					24 (1.5)	454 (3.0)	76 (5.8)	24 (2.0)
Student	N/A	N/A	N/A	N/A	799 (50.5)	937 (62.6)	775 (59.4)	672 (56.2)
Employed					758 (47.9)	507 (33.8)	454 (34.8)	499 (41.8)
**Religion**								
Traditional/other	346 (26.0)	358 (25.7)	275 (22.2)	311 (19.4)	262 (16.6)	172 (11.5)	288 (22.2)	164 (13.7)
Muslim	33 (2.5)	36 (2.6)	19 (1.5)	37 (2.3)	37 (2.3)	24 (1.6)	25 (1.9)	27 (2.3)
Christian	950 (71.5)	1001 (71.8)	945 (76.3)	1252 (78.2)	1282 (81.1)	1301 (86.8)	992 (76.0)	1004 (84.0)

N/A = Variable not available in that survey round

### Trends in median age at first sex using non-parametric and parametric methods

**Fig 2:**
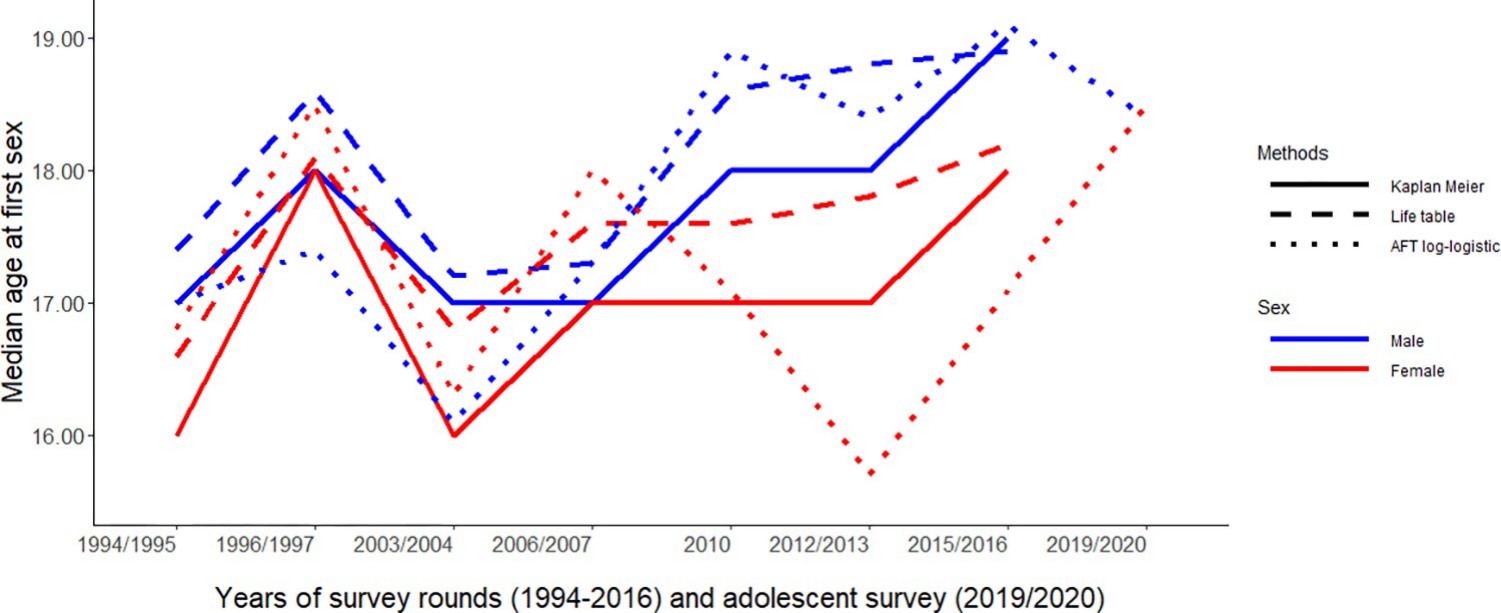
Trends in median age at first sex by sex estimated by parametric and non-parametric methods.

Fig 2 and Table 4 show the trends in median age at first sex. Between 1994 and 2016, the median age at first intercourse increased for both sexes, except for survey round 2 in 1996. For females, the median age at first sexual intercourse increased gradually between 2004 and 2016 from 16.0 to 18.0 when estimated using the Kaplan-Meier method and from 16.8 to 18.2 when estimated using life table methods. Similar trends were observed in males over the same period, with a slightly higher median age at first sexual intercourse (17.0 to 19.0 years using a Kaplan Meier method and 17.2 to 18.9 years using the life table method) compared to females. The Kaplan-Meier and life table estimates were relatively similar. Predicted values from log-logistic accelerated failure time model were used to estimate the median age at first sex in each survey round. The trends are somewhat more erratic for both sexes, however, the values were close to those obtained from Kaplan-Meier and life table methods, with slight difference (lower or higher values) per rounds (Fig 2 and Table 4). In the adolescent survey, the median AFS was not estimated using non- parametric methods (Kaplan-Meier and life table) as less than 50% had intercourse (Table 4).

**Table 4:** Proportion of ever had sex by age and sex, and median age at first sex by sex per each survey rounds.

Years			1994/1995				1996/1997				1999/2000					2003/2004				2006/2007			2010
			Survey 1				Survey 2				Survey 3					Survey 4				Survey 5			Survey 6
**Girls**																							
Ever had sex-N			1023 (75.6)				1198 (85.1)				1088 (80.2)					1381 (83.2)				1134 (66.5)			1123 (65.3)
	N			N				N					N				N				N		
**15**	135		25 (18.5)	44			17 (38.6)	153			38 (24.8)		169			67 (39.6)	326			60 (18.4)	365		70 (19.2)
**16**	163		60 (36.8)	136			64 (47.1)	134			65 (48.5)		196			103 (52.6)	203			55 (27.1)	216		71 (32.9)
**17**	112		62 (55.4)	186			119 (64.0)	140			96 (68.6)		156			114 (73.1)	162			84 (51.9)	179		99 (55.3)
**18**	138		107 (77.5)	142			118 (83.1)	118			103 (87.3)		160			144 (90.0)	142			100 (70.4)	159		117 (73.6)
**19**	117		99 (84.6)	128			120 (93.8)	128			114 (89.1)		176			163 (92.6)	151			131 (86.8)	120		103 (85.8)
**20**	160		153 (95.6)	159			154 (96.9)	143			140 (97.9)		165			157 (95.2)	138			129 (93.5)	132		125 (94.7)
**21**	153		147 (96.1)	138			135 (97.8)	137			132 (96.4)		180			179 (99.4)	155			152 (98.1)	152		147 (96.7)
**22**	127		126 (99.2)	162			159 (98.1)	132			130 (98.5)		143			141 (98.6)	140			138 (98.6)	149		147 (98.7)
**23**	138		135 (97.8)	175			174 (99.4)	145			144 (99.3)		157			155 (98.7)	141			140 (99.3)	121		118 (97.5)
**24**	110		109 (99.1)	138			138 (100.0)	126			126 (100.0)		158			158 (100.0)	146			145 (99.3)	128		126 (98.4)
**Age at first sex**																							
Median (CI’s)-from KM		16.0 (16.0–16.0)			18.0 (17.0–18.0)					N/A				16.0 (16.0–16.0)				17.0 (17.0–18.0)				17.0 (17.0–17.0)	
Median (CI’s)-from LT		16.6 (16.4–16.7)			18.1 (17.8–18.4)									16.8 (16.7–16.9)				17.6 (17.4–17.7)				17.6 (17.4–17.7)	
Median (CI’s)-from AFT		16.8 (16.6–17.0)			18.5 (18.2–18.7)									16.3 (16.1–16.5)				18.0 (17.7–18.3)				17.1 (16.7–17.4)	
**Boys**																							
Ever had sex -N			912 (68.6)				1072 (76.8)				874 (70.5)					1222 (76.4)				1022 (64.6)			721 (48.2)
**15**	173		52 (30.1)	38			12 (31.6)	158			46 (29.1)		200			81 (40.5)	294			120 (40.8)	310		50 (16.1)
**16**	202		80 (39.6)	181			79 (43.6)	135			54 (40.0)		219			122 (55.7)	211			93 (44.1)	214		42 (19.6)
**17**	122		58 (47.5)	223			140 (62.8)	133			68 (51.1)		184			110 (59.8)	216			117 (54.2)	229		84 (36.7)
**18**	133		80 (60.2)	191			144 (75.4)	137			98 (71.5)		159			125 (78.6)	158			97 (61.4)	164		92 (56.1)
**19**	129		105 (81.4)	146			118 (80.8)	129			104 (80.6)		169			146 (86.4)	187			130 (69.5)	125		70 (56.0)
**20**	133		119 (89.5)	153			141 (92.2)	137			118 (86.1)		152			143 (94.1)	142			116 (81.7)	118		87 (73.7)
**21**	134		126 (94.0)	105			99 (94.3)	118			101 (85.6)		141			133 (94.3)	124			109 (87.9)	109		92 (84.4)
**22**	115		109 (94.8)	141			129(91.5)	108			105 (97.2)		120			111 (92.5)	88			83 (94.3)	99		86 (86.9)
**23**	89		85 (95.5)	121			116 (95.9)	101			98 (97.0)		212			117 (96.7)	81			78 (96.3)	70		64 (91.4)
**24**	99		98 (99.0)	96			94 (97.9)	83			82 (98.8)		135			134 (99.3)	80			79 (98.8)	59		54 (91.5)
**Age at first sex**																							
Median (CI’s)-from KM		17.0 (17.0–17.0)				18.0 (18.0–18.0)					N/A				17.0 (17.0–17.0)				17.0 (17.0–17.0)			18.0 (18.0–18.0)	
Median (CI’s)-from LT		17.4 (17.2–17.6)				18.6 (18.4–18.8)									17.2 (17.1–17.4)				17.3 (17.1–17.5)			18.6 (18.5–18.8)	
Median (CI’s)-from AFT		17.0 (16.8–17.2)				17.4 (17.2–17.7)									16.1 (16.0–16.3)				17.3 (17.0–17.6)			18.9 (17.8–20.1)	
**Years**			**2012/2013**				**2015/2016**				**2019/2020**												
			**Survey 7**				**Survey 8**				**Adolescent survey**												
**Girls**																							
Ever had sex-N			867 (58.9)				855 (56.4)				130 (17.9)												
	N			N				N															
**15**	323		24 (7.4)	345			36 (10.4)	110			4 (3.6)												
**16**	155		34 (21.9)	179			31 (17.3)	170			13 (7.6)												
**17**	160		59 (36.9)	137			47 (34.3)	159			25 (15.7)												
**18**	124		84 (67.7)	143			87 (60.8)	141			36 (25.5)												
**19**	141		115 (81.6)	124			94 (75.8)	146			52 (35.6)												
**20**	130		119 (91.5)	133			117 (88.0)																
**21**	116		111 (95.7)	113			106 (93.8)																
**22**	95		94 (98.9)	117			113 (96.6)																
**23**	113		112 (99.1)	119			118 (99.2)																
**24**	116		115 (99.1)	107			106 (99.1)																
**Age at first sex**																							
Median (CI’s)-from KM		17.0 (17.0–18.0)			18.0 (18.0–18.0)				C/E														
Median (CI’s)-from LT		17.8 (17.7–18.0)			18.2 (18.1–18.3)				C/E														
Median (CI’s)-from AFT		15.7 (15.3–16.1)			17.1 (16.7–17.4)				18.5 (17.9–19.1)														
**Boys**																							
Ever had sex-N			644 (49.3)				557 (46.6)				242 (29.5)												
**15**	254		30 (11.8)	261			19 (7.3)	132			21 (15.9)												
**16**	209		50 (23.9)	178			34 (19.1)	148			21 (14.2)												
**17**	174		66 (37.9)	121			38 (31.4)	171			41 (24.0)												
**18**	134		69 (51.5)	158			72 (45.6)	175			62 (35.4)												
**19**	110		66 (60.0)	100			63 (63.0)	194			97 (50.0)												
**20**	122		91 (74.6)	104			84 (80.8)																
**21**	89		77 (86.5)	87			74 (85.1)																
**22**	69		59 (85.5)	67			60 (89.6)																
**23**	70		66 (94.3)	69			65 (94.2)																
**24**	74		70 (94.6)	50			48 (96.0)																
**Age at first sex**																							
Median (CI’s)-from KM		18.0 (18.0–19.0)			19.0 (18.0–19.0)				C/E														
Median (CI’s)-from LT		18.8 (18.6–18.9)			18.9 (18.7–19.0)				C/E														
Median (CI’s)-from AFT		18.4 (17.9–18.9)			19.1 (18.7–19.4)				18.4 (17.7–19.1)														

KM = Kaplan Meier; LT = Life Table; AFT (prediction estimates from log-logistic Accelerated Failure Time model); C/E = Cannot be estimated as less than 50% had sexual intercourse

### Cox, parametric AFT models and RMST in estimating factors associated with AFS

Using data from both sexes combined, univariate analysis were performed for all models in each survey round. All model results have not been presented here, but some model results shown in S1 Table. The estimated results were not that different when fitted with RMST and AFT (using log-logistic and Weibull distribution), in contrast to the results from Cox PH and AFT using exponential distribution. The global test for the proportional hazard model from the Schoenfeld residuals (PH test) shows a violation of the assumption in almost every variable per round as the p-value was less than 0.05. In the univariate analysis for each survey round, some variables were significant in all models (Cox PH, AFT and RMST), while other were significant in one or two models, but not in others. In survey 1, variables including sex, marital status, and religion were significant in all fitted models, while in survey 2 only age was significant in all models. In survey 4, age, sex, marital status, and educational level were significant in all models, while in survey 5 education level and employment status were significant in all models. In survey 6, marital status, educational level and employment status were significant in all models, while in survey 7 and 8, sex, marital status, educational level, and employment status were significant in all models. All significant variables from the univariate analysis were included in the multivariable analysis for each survey round (S2 Table), and the performance of the models was compared (Table 5). Parametric AFT models showed an excellent fit to the data based on AIC/BIC for each round. Additionally, the log-logistic model with the lowest AIC/BIC provided the best fit to the data for survey 1, survey 5, survey 6, survey 7 and survey 8 while the Weibull model provided the best fit to the data for survey 2 and survey 4. In contrast, the Cox PH model showed a poor fit in each round, with higher values of both AIC and BIC (Table 5).

**Table 5:** Comparison of fitness of models based on Akaike information criterion (AIC) and Bayesian Information Criterion (BIC).

	**Survey 1 (1994/1995)**		**Survey 2 (1996/1997)**		**Survey 3 (1999/2000)**		**Survey 4 (2003/2004)**	
**Model**	AIC	BIC	AIC	BIC	AIC	BIC	AIC	BIC
Cox	27001.95	27052.06	31091.56	31131.65			36266.51	36330.83
Log-normal	9553.11	9623.84	12268.65	12304.25			12519.54	12598.49
Log-logistic	9432.13	9502.857	12289.56	12325.17			12302.50	12375.38
Weibull	9656.67	9733.29	12048.55	12101.96			12261.69	12340.64
Exponential	15696.60	15761.44	18449.97	18491.51			20409.21	20476.01
Gamma	9534.94	9599.78	12207.53	12272.81			12428.46	12495.27
Generalized gamma	9517.16	9587.89	12209.55	12252.76			12420.96	12465.84
Gompertz	9975.45	10040.29	12230.45	12295.73			12481.80	12548.60
	**Survey 5 (2006/2007)**		**Survey 6 (2010)**		**Survey 7 (2012/2013)**		**Survey 8 (2015/2016)**	
**Surv**	AIC	BIC	AIC	BIC	AIC	BIC	AIC	BIC
Cox	34903.03	34972.57	25513.91	25602.07	20463.38	20553.83	18868.72	18937.01
Log-normal	12519.61	12587.75	9990.40	10081.38	8563.74	8670.46	7647.89	7730.57
Log-logistic	11129.49	11190.45	9914.67	10005.63	8468.80	8557.73	7539.73	7616.48
Weibull	12731.05	12823.96	9998.36	10089.32	8510.82	8617.54	7648.95	7737.50
Exponential	19937.55	19999.49	15471.88	15544.66	12797.00	12880.01	11962.67	12033.54
Gamma	11213.13	11280.18	9944.57	10023.40	8588.17	8665.24	7604.73	7681.47
Generalized gamma	11209.88	11209.88	9906.39	9991.29	8532.57	8615.58	7573.11	7655.76
Gompertz	11770.44	11837.50	10288.51	10367.34	8799.94	8877.02	7865.68	7942.42

The significant risk factors for AFS in each survey were assessed using multivariable log-logistic AFT model across the eight survey rounds (S2 Table). Compared to those aged 15–19 years, the older age group (20–24 years) reported a significantly older AFS, in the earlier surveys from survey 1 (TR = 1.03; 95% CI: 1.01–1.04) through survey 5 (TR = 1.06, 95% CI: 1.05–1.08). However, in the later surveys there was no significant difference in AFS between the age groups. In later surveys, females reported earlier AFS compared to males from survey 5 (TR = 0.91, 95% CI: 0.84–0.98) through survey 8 ((TR = 0.87, 95% CI: 0.79–0.95). Participants secondary education level showed no significant differences in AFS compared to those with no education in most surveys, except survey 4 (TR = 1.03, 95% CI: 1.01–1.07). Employment status was asked only in the later survey rounds, and those who employed had a significant delay in AFS compared to those unemployed from survey 5 (TR = 0.89, 95% CI: 0.83–0.96) through to survey 8 (TR = 0.89, 95% CI: 0.83–0.96).

### Application of AFT models to adolescent survey data

The adolescent survey was conducted among 1,546 adolescents aged 15–19 years, of whom 820 (53.0%) were males. Most respondents were Christians 1,449 (93.7%) and unemployed 1292 (83.6%). Overall, 1,270 (82.1%) said religion was very important to them and 836 (54.1%) were currently in school. Only 384 (24.8%) owned a mobile phone and 60 (3.9%) had ever drunk alcohol (Table 6).

**Table 6:** Participant characteristics and AFT in estimating factors associated with AFS among adolescent (15–19 years) in Kisesa.

	Total (N = 1546)	Univariate AFT (Log-logistic)	Multivariable AFT (Log-logistic)		
Variable	n (%)	TR	TR	P	95% CI
**Sex**					
Male	820 (53.0)	1	1		
Female	726 (47.0)	1.05***	1.03	0.031	1.01–1.06
**Current in school**					
No	710 (45.9)	1			
Yes	836 (54.1)	1.07***			
**Current level of education studying**					
Not in school	710 (45.9)	1	1		
Primary school	118 (7.6)	0.98	0.98	0.548	0.93–1.04
Secondary school and above	718 (46.4)	1.08***	1.05	0.001	1.02–1.08
**Employment status**					
Unemployed	1292 (83.6)	1	1		
Employed	254 (16.4)	0.92***	0.95	0.001	0.92–0.98
**Religion**					
other/not belong to any	58 (3.8)				
Muslim	39 (2.52)				
Christian	1449 (93.7)				
**How important is religion**					
Not important/don’t know it’s importance	63 (4.1)	1			
Important	213 (13.8)	1.00			
Very Important	1270 (82.2)	1.02			
**Live with mother**					
No	471 (30.5)	1			
Yes	1075 (69.5)	1.00			
**Live with father**					
No	623 (40.3)	1			
Yes	923 (59.7)	1.02			
**Own mobile phone**					
No	1162 (75.2)	1	1		
Yes	384 (24.8)	0.92***	0.94	< .001	0.91–0.96
**Ever used alcohol**					
No	1486 (96.1)	1	1		
Yes	60 (3.9)	0.86***	0.88	< .001	0.84–0.93

***p-value < .001

**p-value .001

*p-value .01; TR = time ratio; AFT = Accelerating Failure Time; AFS = Age at first sex

We examine the applicability of the selected AFT model (using log-logistic and Weibull distributions) from the previous analysis with survey data from co-located adolescents in estimating factors associated with AFS. The log-logistic model with the lowest values of AIC/BIC provided the best fit to the data. The AIC and BIC under log-logistic were 2659.29 and 2701.651, while for Weibull, they were 9656.67 and 9733.29, respectively. The results of the log-logistic AFT model with variables are presented in Table 6. The effect of a variable is to accelerate or decelerate the age at first sex. To better understand this, a time ratio (TR), also called acceleration factor was estimated. A time ratio greater than 1 implies that the variable’s potency effect increases the survival time to first sex (i.e., the event is less likely to occur, as an investigator must wait longer for the event to happen), and a time ratio less than 1 decreases the time until the first sex. After adjusting for other variables, females take a significantly longer time to start sex than males (TR = 1.03, 95% CI: 1.01–1.06), similarly to those reported being currently in secondary education and above compared those who reported not being in school (TR = 1.05 95% CI: 1.02–1.08). Employed adolescents take a shorter time to start sex compared to the unemployed (TR = 0.95, 95% CI: 0.92–0.98), likewise to those who own a mobile phone than those without mobile phone (TR = 0.94, 95% CI: 0.91–0.96) and who ever consumed alcohol than never consumed alcohol (TR = 0.88; 95% CI: 0.84–0.93).

## Discussion

In this study, we evaluate the applicability of survival analysis models in estimating age at first sex (AFS) with minimal bias among the young population. We used the Akaike Information Criteria (AIC) and Bayesian Information Criteria (BIC) as the main performance evaluation measures. All the models could be used to analyse age at first sex, but the Cox PH model underperformed compared to the other models. In every survey round, the Cox PH model had higher AIC/BIC, while the AFT models using the log-logistic distribution were much lower across all rounds except round two and four. Overall, AFT models were the best fit for the analysis of AFS of these data in each round, with lower values in five rounds using log-logistic and lower in two rounds using Weibull distribution. Regardless of the type of distribution, AFT seemed to be the most realistic assumption to use when estimating age at first sex, as it allows an increase or decrease in a hazard (risk), unlike the assumption of constant hazard. For-instance, under log-logistic distribution, AFT allows for hazard to increase and later decrease (i.e., risk of initiating sex increases but decreases with age). Our results are consistent with the study conducted by Mitiku in Ethiopia, which showed the log-logistic AFT model performed better, although their study population consisted only women aged 15–49 [33, 34]. This shows that regardless of whether the data are analysed separately for females and males in different age groups, or combined as in the current study, the AFT model is still observed as the better model when analysing AFS.

The results of the adjusted log-logistic AFT using survey data from adolescents showed that sex, education level, employment status, own a mobile phone, and ever consumed alcohol significantly influenced the timing of first sex. Females and those attending secondary and higher education level had prolonged time to first sex by the factor of TR = 1.03 and TR = 1.05, respectively. Cell phone ownership, employed adolescents, and consumed alcohol shortened time to first sex by the factor of TR = 0.94, TR = 0.95 and TR = 0.88, respectively. The association between these variables and age at first sex has also been found in other studies that only focused on women using other statistical approaches. The underlying reasons for these associations can be multifaceted and influenced by various individual, social, and contextual factors. Adolescents who are employed or consume alcohol may have larger social networks and greater exposure to peers who engage in risky behaviors, including early sexual activity. Peer influence and social pressure can play a significant role in shaping adolescents’ behaviors and decision-making, potentially leading to an earlier age at first sex [35, 36]. Owning a mobile phone and having access to the internet can expose adolescents to a wide range of sexual content, including explicit images, videos, and online communities. This exposure can normalize and desensitize them to sexual behavior, potentially leading to an earlier initiation of sexual activity [37].

The survival function, accounting for censored observations, gives a more efficient and unbiased estimate of AFS, compared to using the proportions who have had sex at any age [8, 30]. Estimates from parametric models allow the estimation of the median AFS while accounting for risk factors, but they are sensitive to the assumed distribution [30, 38]. Estimated values of median age at first sex in both non-parametric and parametric methods were relatively close to each other, with some slight differences (lower or higher values) in each round. Across the eight rounds (over 20 years), the median age at first sex increased for both sexes, although trends for log-logistic AFT are little more erratic. The similarity in median values for the life table and Kaplan Meier was expected since the sample size used in each round (range 2592 to 3287) was large enough to produce similar or close results. The observed differences in trends from the log-logistic AFT model could be due to inconsistencies in multiple responses, thus why we observed fluctuations in the predicted results. However, the large sample size from the same population in each round gives us a good chance to observe different methods applicable to this type of data and choose the best one, which is a good foundation for the method to be used in the future analysis.

## Conclusion and recommendation

This study proposes the accelerated failure time (AFT) model as a better alternative to the popular proportional hazards model in estimating age at first sex. We also fitted this model using seven different distributions for each survey round. Using AIC/BIC, most of the rounds show that AFT with log-logistic distribution is the best model for the data. This was more realistic due to the nature of the data (youth, 15–24 years) and theory of the log-logistic AFT model, which allows the risk of starting sex to increase until the median AFS is reached and then to decrease. A similar study performed in the Bayesian framework, with cross-validation results, showed that the asymmetric log-skew logistic distribution reproduces empirical AFS data better than other commonly used distributions [38]. Although, we did not use a log-skew logistic distribution in this analysis, but it would be instructive to examine this distribution in future analysis using Kisesa data.

For the median age at first sex, values were estimated in each round of the repeated cross- sectional survey, and there is a high possibility that some individuals appeared in more than one round and reported different values. In this analysis, we did not correct for this, so the trend of median age at first sex may be overestimated or underestimated. To estimate trends in AFS accurately in this population, future analysis needs to consider those who appeared in more than one survey round, check for the consistency of the reported AFS, and analyze the data longitudinally. This will allow for an accurate evaluation of significant changes in AFS over time and factors associated with it. Furthermore, the actual reported AFS values were considered in this analysis as completed years from last birthday, but on average, actual AFS values could be 0.5 older than reported values. This could be accounted for in future analysis, or interval-censored observation could be considered around the true AFS to avoid interpreting reported AFS as an exact age, as was conducted by Nguyen [38].

This study showed the overall effects for both sexes combined, demonstrating the statistical model best suited to the analysis of AFS. Interaction effects between sex and all variables in the model were assessed, and for the adolescent survey the only significant effect was for mobile phone ownership. Further analysis of sex-specific effects will be carried out in a future paper, after assessing the consistency of reporting of AFS over time using an analysis of the repeated responses over time by the same individuals. Some of the significant associations for AFS among married and employed young people may be due to reverse causation, which will also be assessed using a longitudinal life-course analysis.

## Supporting information

S1 Questionnaire (DOCX)

S1 Dataset Eight survey rounds and adolescent survey used for this study. (XLSX)

S1 Table Comparison of the results of the fitted Cox, AFT (only exponential, log-logistic and Weibull presented here) models and RMST in univariate analysis for estimating factors associated with AFS. (DOCX)

S2 Table Comparison of the results of fitted Cox, AFT (only log-logistic and exponential distributions presented here) and RMST in multivariable analysis. (DOCX)
