# Supplementary material for: Comparison of survival analysis approaches to modelling age at first sex among youth in Kisesa Tanzania
Source: PLoS One. 2023 Sep 7;18(9):e0289942. doi: 10.1371/journal.pone.0289942 (PMC10484422; doi:10.1371/journal.pone.0289942)
Supplement: S1 Questionnaire — (DOCX) [file pone.0289942.s001.docx]

**Questionnaire:**

TAZAMA Serological survey sub part of questions from questionnaire Sero 1-8

| What sex is this person? | | Male 1 Female 2 | |  |  |
| --- | --- | --- | --- | --- | --- |
| Date of birth  (write 99 if day or month not known, 9999 if year not known) | | └─┴─┘└─┴─┘└─┴─┴─┴─┘  dd mm yyyy | |  |  |
| Age or approximate age | | └─┴─┘years | |  |  |
| Where do you live: Village | | ……………………. | |  |  |
| Which class/level of formal education did you complete? | | UNDER ONE YEAR …..……………….….1  ▐ CLASS 1 - 4 ………...……………...………..2  ▐ ST CLASS 5 - 7/8 .………..…..….………...….. 3  ▐ SECONDARY - 4/6 .…………....……….… 4  ▐ OVER SECONDARY EDUCATION …… 5  ▐ ADULT EDUCATION ..…………………. 6  OTHERS__________________________ 7  Specify | | |  |
| How many years of education did you have? | | number of years completed at each level | | |  |
| Primary | | └─┴─┘years | | |  |
| Secondary (including A level) | | └─┴─┘years | | |  |
| College | | └─┴─┘years | | |  |
| University | | └─┴─┘years | | |  |
| Adult institute | | └─┴─┘years | | |  |
| Religious education | | └─┴─┘years | | |  |
| Other (specify below) | | └─┴─┘years | | |  |
|  | | …………………………… | | |  |
| What is your religion?  Muslim  Catholic  Other Christian (Protestant & Evangelical)  Traditional  None  Other (specify below) | | Circle only one response  1  2  3  4  5  6 | | |  |
| What is your current marital status?  Never married or been in cohabiting union  Monogamously married or cohabiting  Polygamously married or cohabiting  Widowed  Separated or divorced | | Circle only one response  1 never married → q95  2 → q88  3  4 → q88  5 → q88 | | |  |
| Are you doing farm work? | | YES……...……………………….……..1  NO……….…………………………….. 2 |  | | |
| Do you do other work?  If so which ones? | | PUPIL/STUDENT ..………………....…… 1  UNSKILLED LABOURER …....………… 2  SKILLED JOB ……………….…..……….. 3  BUSINESSMAN …………… ……………. 4  FISHERMAN …..…………..…………..… 5  LORRY/TAXI DRIVER ……………….… 6  BAR/GROCERY MAID ……………….. 7  OTHER WORK …………..…………….... 8  OTHER __________________________ 9  Specify |  | | |
| Do you perform any work that helps you or your household earn money?  Yes  No, I am still a student  No, I just look after the house  No, I am too ill to work  No, I am too old to work  No, other reason (specify below) | | **Circle only one response**  1  2  3  4  5  6 |  | | |
|  |  |  |  | | |
| Have you ever-practiced sex? | | YES ……………………………… 1  NO ……………………………….. 2 | | | |
| How old were you when you first married or lived with a sexual partner? (write 99 if not known) | └─┴─┘ years old | | | |  |

**Adolescent survey, sub part of questions from questionnaire**

| Write your age in numbers. | \|___\|___\| | |
| --- | --- | --- |
| Write in numbers your birthdate indicating the day, month and year | \|___\|___\|/\|__\|__\|/\|__\|__\|__\|__\| | |
| What is your gender? | Press 1 if you are a boy | |
|  | Press 2 if you are a girl | |
| What is your denomination? | Press 1 if you are a Catholic Bonyeza | |
|  | Press 2 if you are an African Inland Church (AIC) | |
|  | Press 3 if you belong to a Pentecostal denomination | |
|  | Press 4 if you are an EAGT | |
|  | Press 5 if you are a Sabbath | |
|  | Press 6 if you are a Lutheran (KKKT) | |
|  | Press 7 if you are a non-denominational Christian | |
|  | Press 8 if you are a Muslim | |
|  | Press 9 if you belong to other denominations | |
|  | Press 10 if you don't belong to any denomination | |
| How important is religion to you? | Press 1 if it is very important | |
|  | Press 2 if it is important | |
|  | Press 3 if it is not important | |
|  | Press 4 if you do not know the importance of religion to you | |
| How important is religion to you? | Press 1 if it is very important Bonyeza 1 kama ni muhimu sana | |
|  | Press 2 if it is important | |
|  | Press 3 if it is not important | |
|  | Press 4 if you do not know the importance of religion to you | |
| Are you in school this year? | Press 1 if you are in school this year | |
|  | Press 2 if you are not in school this year | |
| What level of education are you currently studying? | Press 1 if you are in primary school | |
|  | Press 2 if you are in secondary school (form one up to form four) | |
|  | Press 3 if you are in secondary school (form five up to six) | |
|  | Press 4 if you are in University or other college | |
|  | Press 5 if you are in vocational college | |
| Are you currently employed, or do you have a job that pays you any salary or wages? | | Press 1 if Yes |
|  |  | Press 2 if No |

| Do you live with your mother? | Press 1 if Yes |
| --- | --- |
|  | Press 2 if No |
| Do you live with your father? | Press 1 if Yes |
|  | Press 2 if No |

| Do you own a mobile phone? | Press 1 if you have a mobile phone of your own |
| --- | --- |
|  | Press 2 if No |
| Are you married or currently married? | Press 1 if you are married or currently married |
|  | Press 2 if No |
| Have you ever used alcohol | Press 1 if you have ever used alcohol |
|  | Press 2 if you have never-🡪K1 |
| Have you ever had sex? | Press 1 if you have had sex |
|  | Press 2 if No |
| Since you said you already had sex, write down how old you were when you had had sex for the first time? | \|___\|___\| |
