# Supplementary material for: Comparison of survival analysis approaches to modelling age at first sex among youth in Kisesa Tanzania
Source: PLoS One. 2023 Sep 7;18(9):e0289942. doi: 10.1371/journal.pone.0289942 (PMC10484422; doi:10.1371/journal.pone.0289942)
Supplement: S1 Table — (DOCX) [file pone.0289942.s003.docx]

| **S2 Table. Comparison of the final results of fitted Cox, parametric models and RMST in univariate analysis** (P<0.05) | **Surv 8 [2015/2016]** |  | **R. RMST** | |  |  | 0.99**^**^** |  |  | 0.97**^***^** |  |  | 0.97 |  |  | 1.04 | 1.08**^***^** |  |  | 1.03**^*^** |  |  | 0.93**^***^** | 1.07**^***^** |  |  | 1.09**^***^** | 0.87**^***^** |  |  | 1.01 | 1.01 | **^***^**p-value <.001; **^**^**p-value .001; *p-value .01; HR=hazard ratio; ETR=event time ratio; d. RMST=difference in restricted mean survival time; R.RMST=ratio in restricted mean survival time, N/A=Not available in that sero  **^***^**p-value <.001; **^**^**p-value .001; **^*^**p-value .01; HR=hazard ratio; ETR=event time ratio; d.RMST=difference in restricted mean survival time; R.RMST=ratio in restricted mean survival time,N/A=Variable is available in that sero round |
| --- | --- | --- | --- | --- | --- | --- | --- | --- | --- | --- | --- | --- | --- | --- | --- | --- | --- | --- | --- | --- | --- | --- | --- | --- | --- | --- | --- | --- | --- | --- | --- | --- | --- |
|  |  |  | **d.RMST** | |  |  | -0.23**^**^** |  |  | -0.63**^***^** |  |  | -0.55 |  |  | 0.68 | 1.46**^***^** |  |  | 0.49**^*^** |  |  | -1.38**^***^** | 1.24**^***^** |  |  | 1.66**^***^** | -2.60**^***^** |  |  | 0.23 | 0.14 |  |
|  |  | **AFT** | **exponential** | **ETR** |  | 1 | 2.74**^***^** |  | 1 | 1.21**^***^** |  | 1 | 0.95 |  | 1 | 2.51**^***^** | 2.46**^***^** |  | 1 | 0.75**^***^** |  | 1 | 0.94 | 0.58**^***^** |  | 1 | 0.24**^***^** | 1.06 |  | 1 | 1.04 | 1.05 |  |
|  |  |  | **Weibull** |  |  | 1 | 1.01 |  | 1 | 0.96**^***^** |  | 1 | 1.00 |  | 1 | 0.93**^***^** | 0.96**^*^** |  | 1 | 1.01 |  | 1 | 0.97**^*^** | 1.04**^***^** |  | 1 | 1.15**^***^** | 1.01**^***^** |  | 1 | 0.97 | 0.98 |  |
|  |  |  | **Log -logistic** |  |  | 1 | 1.00 |  | 1 | 0.97**^***^** |  | 1 | 1.01 |  | 1 | 0.94**^***^** | 0.95**^*^** |  | 1 | 1.03**^**^** |  | 1 | 0.99 | 1.07**^***^** |  | 1 | 1.11**^***^** | 1.00 |  | 1 | 0.95 | 0.96 |  |
|  |  |  | **Cox** | **HR** |  | 1 | 1.31**^***^** |  | 1 | 1.36**^***^** |  | 1 | 0.96 |  | 1 | 1.99**^***^** | 1.75**^***^** |  | 1 | 0.84 |  | 1 | 1.15 | 0.62**^***^** |  | 1 | 0.31**^***^** | 0.99 |  | 1 | 1.18 | 1.17 |  |
|  |  |  |  |  |  |  |  |  |  |  |  |  |  |  |  |  |  |  |  |  |  |  |  |  |  |  |  |  |  |  |  |  |  |
|  | **Surv 7 [2012/2013]** |  | **R. RMST** | |  |  | 1.00 |  |  | 0.95**^***^** |  |  | 0.99 |  |  | 1.05**^*^** | 1.10**^***^** |  |  | 1.08**^***^** |  |  | 0.93**^***^** | 1.05**^***^** |  |  | 1.09**^***^** | 0.89**^***^** |  |  | 1.03**^*^** | 0.97 |  |
|  |  |  | **d.RMST** | |  |  | 0.05 |  |  | -0.89**^***^** |  |  | -0.10 |  |  | 0.93**^*^** | 1.66**^***^** |  |  | 1.37**^***^** |  |  | -1.25**^***^** | 0.87**^***^** |  |  | 1.51**^***^** | -2.21**^***^** |  |  | 0.49**^*^** | -0.55 |  |
|  |  | **AFT** | **Exponential** | **ETR** |  | 1 | 2.45**^***^** |  | 1 | 1.21**^***^** |  | 1 | 1.00 |  | 1 | 2.41**^***^** | 2.39**^***^** |  | 1 | 0.64**^***^** |  | 1 | 0.91 | 0.70**^***^** |  | 1 | 0.34**^***^** | 1.12 |  | 1 | 0.94 | 1.04 |  |
|  |  |  | **Weibull** |  |  | 1 | 1.01 |  | 1 | 0.94**^***^** |  | 1 | 0.99 |  | 1 | 0.92**^***^** | 0.93**^**^** |  | 1 | 1.05**^***^** |  | 1 | 0.98**^*^** | 1.05**^***^** |  | 1 | 1.13**^***^** | 1.01 |  | 1 | 0.97 | 0.95**^***^** |  |
|  |  |  | **Log- logistic** |  |  | 1 | 0.99 |  | 1 | 0.96**^***^** |  | 1 | 1.00 |  | 1 | 0.92**^***^** | 0.92**^**^** |  | 1 | 1.08**^***^** |  | 1 | 1.01 | 1.08**^***^** |  | 1 | 1.11**^***^** | 1.00 |  | 1 | 1.00 | 0.98 |  |
|  |  |  | **Cox** | **HR** |  | 1 | 1.30**^***^** |  | 1 | 1.51**^***^** |  | 1 | 1.05 |  | 1 | 2.12**^***^** | 2.00**^***^** |  | 1 | 0.63**^***^** |  | 1 | 1.08 | 0.64**^***^** |  | 1 | 0.40**^***^** | 0.99 |  | 1 | 1.04 | 1.27**^**^** |  |
|  |  |  |  |  |  |  |  |  |  |  |  |  |  |  |  |  |  |  |  |  |  |  |  |  |  |  |  |  |  |  |  |  |  |
|  | **Surv 6 [2010]** |  | **R. RMST** | |  |  | 0.99 |  |  | 0.95 |  |  | 0.98**^*^** |  |  | 1.01 | 1.09**^***^** |  |  | 0.97**^*^** |  |  | 0.95**^***^** | 1.04**^***^** |  |  | 1.08**^***^** | 0.92**^***^** |  |  | 1.01 | 1.02 |  |
|  |  |  | **d.RMST** |  |  |  | -0.11 |  |  | -0.89 |  |  | -0.30**^*^** |  |  | 0.19 | 1.53**^***^** |  |  | -0.53**^*^** |  |  | -1.01**^***^** | 0.67**^***^** |  |  | 1.45**^***^** | -1.56**^***^** |  |  | 0.13 | 0.37 |  |
|  |  | **AFT** | **exponential** | **ETR** |  | 1 | 2.15**^***^** |  | 1 | 1.37**^***^** |  | 1 | 1.06 |  | 1 | 2.25**^***^** | 2.15**^***^** |  | 1 | 0.67**^***^** |  | 1 | 0.87**^*^** | 0.76**^***^** |  | 1 | 0.53**^***^** | 1.30**^*^** |  | 1 | 1.33 | 1.04 |  |
|  |  |  | **Weibull** |  |  | 1 | 1.04**^***^** |  | 1 | 0.94**^***^** |  | 1 | 0.98**^**^** |  | 1 | 0.92^***^ | 0.96 |  | 1 | 1.04**^***^** |  | 1 | 0.98**^*^** | 1.04**^***^** |  | 1 | 1.06**^***^** | 0.99 |  | 1 | 0.90**^***^** | 0.97**^**^** |  |
|  |  |  | **Log-logistic** |  |  | 1 | 1.01 |  | 1 | 0.95**^***^** |  | 1 | 0.99 |  | 1 | 0.94**^***^** | 0.95**^*^** |  | 1 | 1.05**^***^** |  | 1 | 1.00 | 1.06**^***^** |  | 1 | 1.03**^***^** | 0.96 |  | 1 | 0.94**^**^** | 0.98 |  |
|  |  |  | **Cox** | **HR** |  | 1 | 1.13**^*^** |  | 1 | 1.63**^***^** |  | 1 | 1.14**^**^** |  | 1 | 2.02**^***^** | 1.66**^***^** |  | 1 | 0.70**^***^** |  | 1 | 1.03 | 0.68**^***^** |  | 1 | 0.58**^***^** | 1.14 |  | 1 | 1.82**^***^** | 1.22**^*^** |  |
|  |  |  |  |  |  |  |  |  |  |  |  |  |  |  |  |  |  |  |  |  |  |  |  |  |  |  |  |  |  |  |  |  |  |
|  | **Surv 5 [2006/2007]** |  | **R. RMST** | |  |  | 0.99 |  |  | 1.00 |  |  | 0.98**^**^** |  |  | N/A | |  |  | 1.03**^**^** |  |  | 0.97**^***^** | 1.01 |  |  | 1.08**^***^** | 0.91**^***^** |  |  | 1 | 1.04**^**^** |  |
|  |  |  | **d.RMST** |  |  |  | -0.11 |  |  | 0.04 |  |  | -0.35**^**^** |  |  | N/A | |  |  | 0.46**^**^** |  |  | -0.47**^***^** | 0.12 |  |  | 1.36**^***^** | -1.74**^***^** |  |  | -0.04 | 0.65**^**^** |  |
|  |  | **AFT** | **exponential** | **ETR** |  | 1 | 1.78**^***^** |  | 1 | 1.02 |  | 1 | 1.09 |  | 1 | 1.78**^***^** | 1.71**^***^** |  | 1 | 0.73**^***^** |  | 1 | 0.73**^***^** | 0.75**^***^** |  | 1 | 0.44**^***^** | 1.1 |  | 1 | 1.07 | 0.96 |  |
|  |  |  | **Weibull** |  |  | 1 | 1.05**^***^** |  | 1 | 1.00 |  | 1 | 0.97**^***^** |  | 1 | 0.97**^***^** | 0.95 |  | 1 | 0.99 |  | 1 | 0.98**^*^** | 1.00 |  | 1 | 1.07**^***^** | 0.99 |  | 1 | 0.95**^*^** | 0.99 |  |
|  |  |  | **Log-logistic** |  |  | 1 | 1.02**^**^** |  | 1 | 1.01**^*^** |  | 1 | 0.99 |  | 1 | 0.97**^***^** | 0.99 |  | 1 | 1.03**^***^** |  | 1 | 1.03**^**^** | 1.05**^***^** |  | 1 | 1.05**^***^** | 0.98 |  | 1 | 1.00 | 1.01 |  |
|  |  |  | **Cox** | **HR** |  | 1 | 1.03 |  | 1 | 1.01 |  | 1 | 1.16**^***^** |  | 1 | 1.49**^***^** | 1.36**^*^** |  | 1 | 0.87**^*^** |  | 1 | 0.91 | 0.77**^***^** |  | 1 | 0.52**^***^** | 1.11 |  | 1 | 1.25 | 1.03 |  |
|  |  |  |  |  |  |  |  |  |  |  |  |  |  |  |  |  |  |  |  |  |  |  |  |  |  |  |  |  |  |  |  |  |  |
|  | **Surv 4 [2003/2004]** |  | **R. RMST** | |  |  | 1.04**^***^** |  |  | 0.98**^**^** |  |  | 0.99 |  |  | 1.06**^***^** | 1.03**^***^** |  |  | 1.04**^***^** |  |  | 0.96**^***^** | 1.00 |  |  | N/A |  |  |  | 1.01 | 1.01 |  |
|  |  |  | **d.RMST** |  |  |  | 0.70**^***^** |  |  | -0.31**^**^** |  |  | -0.12 |  |  | 0.86**^***^** | 0.52**^***^** |  |  | 0.66**^***^** |  |  | -0.78**^***^** | -0.07 |  |  | N/A |  |  |  | 0.10 | 0.10 |  |
|  |  | **AFT** | **exponential** | **ETR** |  | 1 | 1.36**^***^** |  | 1 | 1.09^*^ |  | 1 | 1.11**^**^** |  | 1 | 1.41**^***^** | 1.44**^**^** |  | 1 | 0.85**^**^** |  | 1 | 0.85**^**^** | 0.85**^*^** |  |  | N/A |  |  | 1 | 1.22 | 1.08 |  |
|  |  |  | **Weibull** |  |  | 1 | 1.06**^***^** |  | 1 | 0.97**^***^** |  | 1 | 1.00 |  | 1 | 0.96**^***^** | 0.95**^***^** |  | 1 | 0.77**^***^** |  | 1 | 1.03**^***^** | 1.08**^***^** |  |  | N/A |  |  | 1 | 0.99 | 0.99 |  |
|  |  |  | **Log-logistic** |  |  | 1 | 1.05**^***^** |  | 1 | 0.98 |  | 1 | 1.00 |  | 1 | 0.98**^**^** | 0.97**^*^** |  | 1 | 1.04**^***^** |  | 1 | 1.04**^***^** | 1.09**^***^** |  |  | N/A |  |  | 1 | 1.00 | 1.00 |  |
|  |  |  | **Cox** | **HR** |  | 1 | 0.75**^***^** |  | 1 | 1.27**^***^** |  | 1 | 1.04 |  | 1 | 1.39**^***^** | 1.62**^***^** |  | 1 | 0.74**^***^** |  | 1 | 0.77**^***^** | 0.56**^***^** |  |  | N/A |  |  | 1 | 1.13 | 1.06 |  |
|  |  |  |  |  |  |  |  |  |  |  |  |  |  |  |  |  |  |  |  |  |  |  |  |  |  |  |  |  |  |  |  |  |  |
|  | **S Surv 2 [1996/1997]** |  | **R. RMST** | |  |  | 1.15**^***^** |  |  | 0.99 |  |  | 0.99**^*^** |  |  | 0.95**^**^** | 0.95**^***^** |  |  | 0.99 |  |  | 0.98 | 1.01 |  |  | N/A |  |  |  | 1.00 | 0.99 |  |
|  |  |  | **d.RMST** |  |  |  | 2.39**^***^** |  |  | -0.14 |  |  | -0.27**^*^** |  |  | -0.92**^**^** | -0.86**^***^** |  |  | -0.15 |  |  | -0.38 | 0.20 |  |  | N/A |  |  |  | -0.08 | -0.14 |  |
|  |  | **AFT** | **exponential** | **ETR** |  | 1 | 1.27**^***^** |  | 1 | 1.11**^*^** |  | 1 | 1.06 |  | 1 | 1.33**^***^** | 1.28**^*^** |  | 1 | 0.94 |  | 1 | 0.94 | 1.04 |  |  | N/A |  |  | 1 | 1.18 | 1.13**^*^** |  |
|  |  |  | **Weibull** |  |  | 1 | 1.16**^***^** |  | 1 | 0.99**^*^** |  | 1 | 0.98**^**^** |  | 1 | 0.84**^***^** | 0.71**^***^** |  | 1 | 1.00 |  | 1 | 1.00 | 1.00 |  |  | N/A |  |  | 1 | 1.00 | 0.99 |  |
|  |  |  | **Log-logistic** |  |  | 1 | 1.10**^***^** |  | 1 | 0.97**^**^** |  | 1 | 0.97**^**^** |  | 1 | 1.00 | 1.02 |  | 1 | 1.00 |  | 1 | 1.00 | 1.01 |  |  | N/A |  |  | 1 | 0.98 | 0.99 |  |
|  |  |  | **Cox** | **HR** |  | 1 | 0.47**^***^** |  | 1 | 1.13**^**^** |  | 1 | 1.13**^**^** |  | 1 | 0.95 | 0.81**^*^** |  | 1 | 0.99 |  | 1 | 0.99 | 1.03 |  |  | N/A |  |  | 1 | 1.04 | 1.11 |  |
|  |  |  |  |  |  |  |  |  |  |  |  |  |  |  |  |  |  |  |  |  |  |  |  |  |  |  |  |  |  |  |  |  |  |
|  | **Surv 1 [1994/1995]** |  | **R. RMST** |  |  |  | 0.95**^***^** |  |  | 0.97**^***^** |  |  | 0.98**^**^** |  |  | 1.06**^**^** | 1.08**^***^** |  |  | 1.01 |  |  | 0.97 | 1.00 |  |  | N/A |  |  |  | 1.01 | 1.04**^*^** |  |
|  |  |  | **d.RMST** |  |  |  | -0.97**^***^** |  |  | -0.48**^***^** |  |  | -0.29**^**^** |  |  | 0.89 | 1.34 |  |  | 0.22 |  |  | -0.51 | -0.06 |  |  | N/A |  |  |  | 0.23 | 0.69**^*^** |  |
|  |  | **AFT** | **exponential** | **ETR** |  | 1 | 1.77**^***^** |  | 1 | 1.12**^*^** |  | 1 | 1.13**^*^** |  | 1 | 1.69**^***^** | 1.69**^***^** |  | 1 | 0.93 |  | 1 | 0.93 | 1.04 |  |  | N/A |  |  | 1 | 1.31 | 1.14**^*^** |  |
|  |  |  | **Weibull** |  |  | 1 | 1.00 |  | 1 | 0.96**^***^** |  | 1 | 0.98**^**^** |  | 1 | 0.92**^***^** | 0.95**^***^** |  | 1 | 1.01 |  | 1 | 1.00 | 1.04**^*^** |  |  | N/A |  |  | 1 | 0.95**^**^** | 0.98**^*^** |  |
|  |  |  | **Log-logistic** |  |  | 1 | 1.00**^***^** |  | 1 | 0.97**^***^** |  | 1 | 0.99 |  | 1 | 0.95**^***^** | 0.94**^***^** |  | 1 | 1.01 |  | 1 | 1.01 | 1.05**^**^** |  |  | N/A |  |  | 1 | 0.93**^*^** | 0.99 |  |
|  |  |  | **Cox** | **HR** |  | 1 | 1.34**^***^** |  | 1 | 1.31**^***^** |  | 1 | 1.16**^**^** |  | 1 | 2.00**^***^** | 1.90**^***^** |  | 1 | 0.92 |  | 1 | 0.93 | 0.79 |  |  | N/A |  |  | 1 | 1.52**^**^** | 1.16**^*^** |  |
|  |  |  | **Variables** |  | **Age group (years)** | 15-19 | 20-24 | **Sex** | Male | Female | **Residence** | Rural | peri-urban/urban | **Marital status** | Never married | Monogamous/ polygamous | Widow/Separated | **Formal education** | No | Yes | **Level of formal education** | No education | Primary education (1-4/5-7) | Secondary/adults/other education | **Employment status** | Unemployed | Student | Employed | **Religion** | Traditional/other | Muslim | Christian |  |
