# Supplementary material for: Comparison of survival analysis approaches to modelling age at first sex among youth in Kisesa Tanzania
Source: PLoS One. 2023 Sep 7;18(9):e0289942. doi: 10.1371/journal.pone.0289942 (PMC10484422; doi:10.1371/journal.pone.0289942)
Supplement: S2 Table — (DOCX) [file pone.0289942.s004.docx]

| **Comparison of the results of fitted Cox, AFT (only 2 distributions presented here) and RMST in multivariable analysis** | | | | | | | | | | | | | | | | | |
| --- | --- | --- | --- | --- | --- | --- | --- | --- | --- | --- | --- | --- | --- | --- | --- | --- | --- |
| **Surv 1 [1994/1995]** | | | | | | | | | | | | | | | | | |
|  | |  |  |  |  | **AFT** | | | | | | |  |  | **RMST** | | |
|  | | **Cox** | | |  | **Log-logistic** | | |  |  | **Exponential** | | |  |  | | |
|  | | **HR** | **P** | **95% CI** |  | **TR** | **P** | **95% CI** |  |  | **TR** | **P** | **95% CI** |  | **exp(R.RMST)** | **P** | **95% CI** |
| **Age group (years)** |  | |  |  |  |  |  |  |  |  |  |  |  |  |  |  |  |
| 15-19 | 1 | |  |  |  | 1 |  |  |  |  | 1 |  |  |  |  |  |  |
| 20-24 | 0.96 | | 0.405 | 0.86-1.06 |  | 1.03 | <.001 | 1.01-1.04 |  |  | 1.71 | <.001 | 1.48-1.97 |  | 1.12 | <.001 | 1.10-1.13 |
| **Sex** |  | |  |  |  |  |  |  |  |  |  |  |  |  |  |  |  |
| Male | 1 | |  |  |  | 1 |  |  |  |  | 1 |  |  |  |  |  |  |
| Female | 0.83 | | 0.004 | 0.73-0.94 |  | 1.02 | 0.033 | 1.00-1.03 |  |  | 0.93 | 0.350 | 0.80-1.08 |  | 0.97 | <.001 | 0.96-0.98 |
| **Residence** |  | |  |  |  |  |  |  |  |  |  |  |  |  |  |  |  |
| Rural | 1 | |  |  |  | …. | ….. | …... |  |  | 1 |  |  |  |  |  |  |
| peri-urban/urban | 1.12 | | 0.024 | 1.01-1.23 |  |  |  |  |  |  | 1.09 | 0.089 | 0.99-1.20 |  | 1.00 | 0.672 | 0.99-1.02 |
| **Marital status** |  | |  |  |  |  |  |  |  |  |  |  |  |  |  |  |  |
| Never married | 1 | |  |  |  | 1 |  |  |  |  | 1 |  |  |  |  |  |  |
| Monogamous/ polygamous | 1.45 | | <.001 | 1.21-1.75 |  | 0.98 | 0.071 | 0.96-1.00 |  |  | 1.16 | 0.131 | 0.96-1.40 |  | 0.99 | 0.884 | 0.91-1.08 |
| Widow/Separated | 1.13 | | 0.711 | 0.60-2.11 |  | 0.98 | 0.682 | 0.90-1.07 |  |  | 1.09 | 0.786 | 0.58-2.04 |  | 1.03 | 0.006 | 1.01-1.05 |
| **Level of formal education** |  | |  |  |  |  |  |  |  |  |  |  |  |  |  |  |  |
| No education |  | |  |  |  | 1 |  |  |  |  |  |  |  |  |  |  |  |
| Primary education (1-4/5-7) | …. | | ….. | …... |  | 1.01 | 0.249 | 0.99-1.03 |  |  | …. | ….. | …... |  | …. | ….. | …... |
| Secondary or higher education |  | |  |  |  | 1.03 | 0.088 | 1.00-1.07 |  |  |  |  |  |  |  |  |  |
| **Employment status** |  | |  |  |  |  |  |  |  |  |  |  |  |  |  |  |  |
| Unemployed |  | |  |  |  |  |  |  |  |  |  |  |  |  |  |  |  |
| Student |  | | N/A |  |  |  | N/A |  |  |  |  | N/A |  |  |  | N/A |  |
| Employed |  | |  |  |  |  |  |  |  |  |  |  |  |  |  |  |  |
| **Religion** |  | |  |  |  |  |  |  |  |  |  |  |  |  |  |  |  |
| Traditional/other | 1 | |  |  |  | 1 |  |  |  |  | 1 |  |  |  |  |  |  |
| Muslim | 1.30 | | 0.072 | 0.98-1.72 |  | 0.96 | 0.025 | 0.93-0.995 | |  | 1.18 | 0.241 | 0.89-1.57 |  | 1.07 | 0.001 | 1.03-1.12 |
| Christian | 1.08 | | 0.201 | 0.96-1.22 |  | 0.99 | 0.23 | 0.98-1.01 |  |  | 1.11 | 0.108 | 0.98-1.25 |  | 1.03 | 0.346 | 0.97-1.08 |
| **Sex: Age group (years)** |  | |  |  |  |  |  |  |  |  |  |  |  |  |  |  |  |
| Male*15-19 |  | |  |  |  |  |  |  |  |  | 1 |  |  |  |  |  |  |
| Female*20-24 |  | |  |  |  |  |  |  |  |  | 0.77 | 0.012 | 0.62-0.94 |  |  |  |  |
| **Sex: Marital status** |  | |  |  |  |  |  |  |  |  |  |  |  |  |  |  |  |
| Male*Never married | 1 | |  |  |  | 1 |  |  |  |  | 1 |  |  |  |  |  |  |
| Female*Monogamous/ polygamous | 1.72 | | <.001 | 1.38-2.14 |  | 0.94 | <.001 | 0.92-0.97 |  |  | 1.48 | 0.002 | 1.16-1.88 |  |  |  |  |
| Female*Widow/Separated | 2.16 | | 0.029 | 1.08-4.29 |  | 0.93 | 0.122 | 0.84-1.02 |  |  | 1.52 | 0.237 | 0.76-3.05 |  |  |  |  |
| **Surv 2 [1996/1997]** | | | | | | | | | | | | | | | | | |
| **Age group (years)** |  | |  |  |  |  |  |  |  |  |  |  |  |  |  |  |  |
| 15-19 | 1 | |  |  |  | 1 |  |  |  |  | 1 |  |  |  |  |  |  |
| 20-24 | 0.44 | | <.001 | 0.40-0.50 |  | 1.12 | <.001 | 1.10-1.15 |  |  | 1.15 | 0.003 | 1.05-1.27 |  | 0.64 | <.001 | 0.61-0.66 |
| **Sex** |  | |  |  |  |  |  |  |  |  |  |  |  |  |  |  |  |
| Male | 1 | |  |  |  | 1 |  |  |  |  | 1 |  |  |  |  |  |  |
| Female | 1.01 | | 0.902 | 0.89-1.14 |  | 0.98 | 0.060 | 0.96-1.00 |  |  | 0.97 | 0.570 | 0.88-1.07 |  | 1.08 | <.001 | 1.04-1.12 |
| **Residence** |  | |  |  |  |  |  |  |  |  |  |  |  |  |  |  |  |
| Rural | 1 | |  |  |  | 1 |  |  |  |  | …. | ….. | …... |  |  |  |  |
| peri-urban/urban | 1.13 | | 0.006 | 1.04-1.23 |  | 0.98 | 0.001 | 0.96-0.99 |  |  |  |  |  |  | 1.05 | 0.012 | 1.01-1.09 |
| **Marital status** |  | |  |  |  |  |  |  |  |  |  |  |  |  |  |  |  |
| Never married | 1 | |  |  |  |  |  |  |  |  | 1 |  |  |  |  |  |  |
| Monogamous/ polygamous | 0.97 | | 0.709 | 0.81-1.15 |  | …. | ….. | …... |  |  | 1.26 | <.001 | 1.13-1.41 |  | 1 | 0.979 | 0.87-1.15 |
| Widow/Separated | 0.78 | | 0.217 | 0.53-1.16 |  |  |  |  |  |  | 1.19 | 0.092 | 0.97-1.46 |  | 0.99 | 0.764 | 0.94-1.05 |
| **Level of formal education** |  | |  |  |  |  |  |  |  |  |  |  |  |  |  |  |  |
| No education |  | |  |  |  |  |  |  |  |  |  |  |  |  |  |  |  |
| Primary education (1-4/5-7) | …. | | ….. | …... |  | …. | ….. | …... |  |  | …. | ….. | …... |  | …. | ….. | …... |
| Secondary or higher education |  | |  |  |  |  |  |  |  |  |  |  |  |  |  |  |  |
| **Employment status** |  | |  |  |  |  |  |  |  |  |  |  |  |  |  |  |  |
| Unemployed |  | |  |  |  |  |  |  |  |  |  |  |  |  |  |  |  |
| Student | N/A | | N/A | N/A |  | N/A | N/A | N/A |  |  | N/A | N/A | N/A |  | N/A | N/A | N/A |
| Employed |  | |  |  |  |  |  |  |  |  |  |  |  |  |  |  |  |
| **Religion** |  | |  |  |  |  |  |  |  |  |  |  |  |  |  |  |  |
| Traditional/other |  | |  |  |  |  |  |  |  |  | 1 |  |  |  |  |  |  |
| Muslim | …. | | ….. | …... |  | …. | ….. | …... |  |  | 1.12 | 0.403 | 0.86-1.46 |  | …. | ….. | …... |
| Christian |  | |  |  |  |  |  |  |  |  | 1.10 | 0.087 | 0.99-1.24 |  |  |  |  |
| **Sex: Age group (years)** |  | |  |  |  |  |  |  |  |  |  |  |  |  |  |  |  |
| Male*15-19 |  | |  |  |  | 1 |  |  |  |  |  |  |  |  |  |  |  |
| Female*20-24 |  | |  |  |  | 0.96 | 0.004 | 0.93-0.99 |  |  |  |  |  |  |  |  |  |
| **Sex: Marital status** |  | |  |  |  |  |  |  |  |  |  |  |  |  |  |  |  |
| Male*Never married | 1 | |  |  |  |  |  |  |  |  |  |  |  |  |  |  |  |
| Female*Monogamous/ polygamous | 1.30 | | 0.015 | 1.05-1.60 |  |  |  |  |  |  |  |  |  |  |  |  |  |
| Female*Widow/Separated | 1.52 | | 0.070 | 0.97-2.40 |  |  |  |  |  |  |  |  |  |  |  |  |  |
| **Surv 4 [2003/2004]** | | | | | | | | | | | | | | | | | |
| **Age group (years)** |  | |  |  |  |  |  |  |  |  |  |  |  |  |  |  |  |
| 15-19 | 1 | |  |  |  | 1 |  |  |  |  | 1 |  |  |  |  |  |  |
| 20-24 | 0.58 | | <.001 | 0.52-0.63 |  | 1.07 | <.001 | 1.06-1.08 |  |  | 1.31 | <.001 | 1.16-1.48 |  | 1.1 | <.001 | 1.09-1.11 |
| **Sex** |  | |  |  |  |  |  |  |  |  |  |  |  |  |  |  |  |
| Male | 1 | |  |  |  | 1 |  |  |  |  | 1 |  |  |  |  |  |  |
| Female | 1.17 | | 0.182 | 0.93-1.49 |  | 0.99 | 0.473 | 0.96-1.02 |  |  | 0.94 | 0.314 | 0.83-1.06 |  | 0.99 | 0.085 | 0.98-1.00 |
| **Residence** |  | |  |  |  |  |  |  |  |  |  |  |  |  |  |  |  |
| Rural | 1 | |  |  |  | ….. | ….. | ….. |  |  | 1 |  |  |  | …. | ….. | …... |
| peri-urban/urban | 1.12 | | 0.005 | 1.03-1.21 |  |  |  |  |  |  | 1.10 | 0.017 | 1.02-1.19 |  |  |  |  |
| **Marital status** |  | |  |  |  |  |  |  |  |  |  |  |  |  |  |  |  |
| Never married | 1 | |  |  |  | 1 |  |  |  |  | 1 |  |  |  |  |  |  |
| Monogamous/ polygamous | 1.46 | | <.001 | 1.24-1.73 |  | 0.96 | 0.003 | 0.94-0.99 |  |  | 1.16 | 0.085 | 0.98-1.37 |  | 1.05 | <.001 | 1.02-1.08 |
| Widow/Separated | 1.03 | | 0.943 | 0.51-2.06 |  | 1.02 | 0.611 | 0.93-1.12 |  |  | 1.04 | 0.918 | 0.52-2.09 |  | 1.03 | <.001 | 1.02-1.04 |
| **Level of formal education** |  | |  |  |  |  |  |  |  |  |  |  |  |  |  |  |  |
| No education | 1 | |  |  |  | 1 |  |  |  |  | 1 |  |  |  |  |  |  |
| Primary education (1-4/5-7) | 0.88 | | 0.198 | 0.73-1.07 |  | 1.02 | 0.167 | 0.99-1.04 |  |  | 0.88 | 0.024 | 0.78-0.98 |  | 0.96 | <.001 | 0.94-0.97 |
| Secondary or higher education | 0.80 | | 0.075 | 0.62-1.02 |  | 1.03 | 0.038 | 1.01-1.07 |  |  | 0.90 | 0.256 | 0.76-1.08 |  | 1.01 | 0.422 | 0.99-1.02 |
| **Employment status** |  | |  |  |  |  |  |  |  |  |  |  |  |  |  |  |  |
| Unemployed |  | |  |  |  |  |  |  |  |  |  |  |  |  |  |  |  |
| Student | N/A | | N/A | N/A |  | N/A | N/A | N/A |  |  | N/A | N/A | N/A |  | N/A | N/A | N/A |
| Employed |  | |  |  |  |  |  |  |  |  |  |  |  |  |  |  |  |
| **Religion** |  | |  |  |  |  |  |  |  |  |  |  |  |  |  |  |  |
| Traditional/other |  | |  |  |  |  |  |  |  |  |  |  |  |  |  |  |  |
| Muslim | …. | | ….. | …... |  | …. | ….. | …... |  |  | …. | ….. | …... |  | …. | ….. | …... |
| Christian |  | |  |  |  |  |  |  |  |  |  |  |  |  |  |  |  |
| **Sex: Age group (years)** |  | |  |  |  |  |  |  |  |  |  |  |  |  |  |  |  |
| Male*15-19 |  | |  |  |  |  |  |  |  |  | 1 |  |  |  |  |  |  |
| Female*20-24 |  | |  |  |  |  |  |  |  |  | 0.81 | 0.022 | 0.68-0.97 |  |  |  |  |
| **Sex: Marital status** |  | |  |  |  |  |  |  |  |  |  |  |  |  |  |  |  |
| Male*Never married | 1 | |  |  |  | 1 |  |  |  |  | 1 |  |  |  |  |  |  |
| Female*Monogamous/ polygamous | 1.21 | | 0.054 | 1.00-1.488 | | 0.99 | 0.325 | 0.96-1.01 |  |  | 1.3 | 0.018 | 1.05-1.61 |  |  |  |  |
| Female*Widow/Separated | 2.32 | | 0.027 | 1.01-4.90 |  | 0.90 | 0.035 | 0.82-0.99 |  |  | 1.49 | 0.298 | 0.70-3.16 |  |  |  |  |
| **Sex: Level of formal education** |  | |  |  |  |  |  |  |  |  |  |  |  |  |  |  |  |
| Male*No education | 1 | |  |  |  | 1 |  |  |  |  |  |  |  |  |  |  |  |
| Female*Primary education (1-4/5-7) | 0.81 | | 0.074 | 0.64-1.02 |  | 1.02 | 0.160 | 0.99-1.05 |  |  |  |  |  |  |  |  |  |
| Female* Secondary or higher education | 0.60 | | 0.004 | 0.42-0.85 |  | 1.05 | 0.016 | 1.01-1.10 |  |  |  |  |  |  |  |  |  |
| **Surv 5 [2006/2007]** | | | | | | | | | | | | | | | | | |
| **Age group (years)** |  | |  |  |  |  |  |  |  |  |  |  |  |  |  |  |  |
| 15-19 | …. | | ….. | …... |  | 1 |  |  |  |  | 1 |  |  |  |  |  |  |
| 20-24 |  | |  |  |  | 1.06 | <.001 | 1.05-1.08 |  |  | 1.16 | 0.001 | 1.06-1.27 |  | 1.06 | <.001 | 1.05-1.07 |
| **Sex** |  | |  |  |  |  |  |  |  |  |  |  |  |  |  |  |  |
| Male | 1 | |  |  |  | 1 |  |  |  |  | 1 |  |  |  | …. | ….. | …... |
| Female | 2.39 | | 0.013 | 1.20-4.75 |  | 0.91 | 0.012 | 0.84-0.98 |  |  | 2.07 | 0.033 | 1.06-4.04 |  |  |  |  |
| **Residence** |  | |  |  |  |  |  |  |  |  |  |  |  |  |  |  |  |
| Rural | 1 | |  |  |  | …. | ….. | …... |  |  | 1 |  |  |  |  |  |  |
| peri-urban/urban | 1.17 | | <.001 | 1.07-1.27 |  |  |  |  |  |  | 1.09 | 0.05 | 1.00-1.18 |  | 1.00 | 0.869 | 0.99-1.02 |
| **Marital status** |  | |  |  |  |  |  |  |  |  |  |  |  |  |  |  |  |
| Never married |  | |  |  |  |  |  |  |  |  |  |  |  |  |  |  |  |
| Monogamous/ polygamous | …. | | ….. | …... |  | …. | ….. | …... |  |  | N/A | N/A | N/A |  | N/A | N/A | N/A |
| Widow/Separated |  | |  |  |  |  |  |  |  |  |  |  |  |  |  |  |  |
| **Level of formal education** |  | |  |  |  |  |  |  |  |  |  |  |  |  |  |  |  |
| No education | 1 | |  |  |  | 1 |  |  |  |  | 1 |  |  |  |  |  |  |
| Primary education (1-4/5-7) | 1.19 | | 0.081 | 0.98-1.43 |  | 1.01 | 0.131 | 1.00-1.03 |  |  | 1.00 | 0.926 | 0.89-1.11 |  | 0.99 | 0.199 | 0.97-1.01 |
| Secondary or higher education | 1.32 | | 0.016 | 1.05-1.65 |  | 1.01 | 0.237 | 0.99-1.03 |  |  | 1.26 | 0.001 | 1.09-1.45 |  | 1.01 | 0.367 | 0.99-1.03 |
| **Employment status** |  | |  |  |  |  |  |  |  |  |  |  |  |  |  |  |  |
| Unemployed | 1 | |  |  |  | 1 |  |  |  |  | 1 |  |  |  |  |  |  |
| Student | 1.14 | | 0.699 | 0.59-2.22 |  | 0.97 | 0.397 | 0.90-1.04 |  |  | 1.10 | 0.773 | 0.57-2.14 |  | 1 | 0.859 | 0.99-1.02 |
| Employed | 2.17 | | 0.014 | 1.18-4.40 |  | 0.89 | 0.003 | 0.83-0.96 |  |  | 2.05 | 0.033 | 1.06-3.97 |  | 1.01 | 0.303 | 0.99-1.03 |
| **Religion** |  | |  |  |  |  |  |  |  |  |  |  |  |  |  |  |  |
| Traditional/other | 1 | |  |  |  |  |  |  |  |  |  |  |  |  |  |  |  |
| Muslim | 1.35 | | 0.035 | 1.02-1.79 |  | …. | ….. | …... |  |  | …. | ….. | …... |  | 0.99 | 0.671 | 0.97-1.02 |
| Christian | 1.10 | | 0.201 | 0.95-1.27 |  |  |  |  |  |  |  |  |  |  | 1.04 | 0.007 | 1.01-1.07 |
| **Sex: Level of formal education** |  | |  |  |  |  |  |  |  |  |  |  |  |  |  |  |  |
| Male*No education | 1 | |  |  |  |  |  |  |  |  |  |  |  |  |  |  |  |
| Female*Primary education (1-4/5-7) | 0.85 | | 0.154 | 0.67-1.06 |  |  |  |  |  |  |  |  |  |  |  |  |  |
| Female* Secondary or higher education | 0.73 | | 0.038 | 0.54-0.98 |  |  |  |  |  |  |  |  |  |  |  |  |  |
| **Sex: Employment status** |  | |  |  |  |  |  |  |  |  |  |  |  |  |  |  |  |
| Male*Unemployed | 1 | |  |  |  | 1 |  |  |  |  | 1 |  |  |  |  |  |  |
| Female*Student | 0.36 | | 0.004 | 0.18-0.72 |  | 1.14 | <.001 | 1.06-1.24 |  |  | 0.28 | 0.0003 | 0.14-0.56 |  |  |  |  |
| Female*Employed | 0.47 | | 0.026 | 0.24-0.91 |  | 1.11 | 0.008 | 1.03-1.20 |  |  | 0.52 | 0.055 | 0.26-1.01 |  |  |  |  |
| **Surv 6 [2010]** | | | | | | | | | | | | | | | | | |
| **Age group (years)** |  | |  |  |  |  |  |  |  |  |  |  |  |  |  |  |  |
| 15-19 | 1 | |  |  |  | …. | ….. | …... |  |  | 1 |  |  |  | …. | ….. | …... |
| 20-24 | 0.85 | | 0.059 | 0.71-1.01 |  |  |  |  |  |  | 1.77 | <.001 | 1.50-2.08 |  |  |  |  |
| **Sex** |  | |  |  |  |  |  |  |  |  |  |  |  |  |  |  |  |
| Male | 1 | |  |  |  | 1 |  |  |  |  | 1 |  |  |  |  |  |  |
| Female | 4.48 | | <.001 | 2.41-8.31 |  | 0.89 | 0.001 | 0.83-0.95 |  |  | 2.57 | 0.002 | 1.43-4.59 |  | 0.95 | 0.268 | 0.87-1.04 |
| **Residence** |  | |  |  |  |  |  |  |  |  |  |  |  |  |  |  |  |
| Rural | 1 | |  |  |  | …. | ….. | …... |  |  | …. | ….. | …... |  |  |  |  |
| peri-urban/urban | 1.16 | | 0.006 | 1.04-1.28 |  |  |  |  |  |  |  |  |  |  | 0.99 | 0.328 | 0.98-1.01 |
| **Marital status** |  | |  |  |  |  |  |  |  |  |  |  |  |  |  |  |  |
| Never married | 1 | |  |  |  | 1 |  |  |  |  | 1 |  |  |  |  |  |  |
| Monogamous/ polygamous | 1.58 | | <.001 | 1.38-1.82 |  | 0.99 | 0.690 | 0.96-1.03 |  |  | 1.33 | <.001 | 1.16-1.54 |  | 0.95 | 0.217 | 0.88-1.03 |
| Widow/Separated | 1.41 | | 0.034 | 1.03-1.94 |  | 1.16 | 0.054 | 1.00-1.34 |  |  | 1.30 | 0.110 | 0.94-1.79 |  | 1.06 | 0.104 | 0.99-1.13 |
| **Level of formal education** |  | |  |  |  |  |  |  |  |  |  |  |  |  |  |  |  |
| No education | 1 | |  |  |  | 1 |  |  |  |  | 1 |  |  |  |  |  |  |
| Primary education (1-4/5-7) | 1.27 | | 0.029 | 1.03-1.58 |  | 1.00 | 0.638 | 0.98-1.01 |  |  | 1.05 | 0.381 | 0.94-1.18 |  | 1.07 | 0.013 | 1.02-1.13 |
| Secondary or higher education | 1.26 | | 0.062 | 0.99-1.62 |  | 1.02 | 0.107 | 1.00-1.04 |  |  | 1.43 | <.001 | 1.23-1.67 |  | 0.92 | 0.002 | 0.88-0.97 |
| **Employment status** |  | |  |  |  |  |  |  |  |  |  |  |  |  |  |  |  |
| Unemployed | 1 | |  |  |  | 1 |  |  |  |  | 1 |  |  |  |  |  |  |
| Student | 1.48 | | 0.156 | 0.86-2.56 |  | 0.95 | 0.147 | 0.90-1.02 |  |  | 1.01 | 0.963 | 0.59-1.74 |  | 1.13 | <.001 | 1.07-1.19 |
| Employed | 2.21 | | 0.004 | 1.29-3.80 |  | 0.92 | 0.014 | 0.87-0.98 |  |  | 1.70 | 0.054 | 0.99-2.91 |  | 0.88 | <.001 | 0.84-0.93 |
| **Religion** |  | |  |  |  |  |  |  |  |  |  |  |  |  |  |  |  |
| Traditional/other | 1 | |  |  |  | 1 |  |  |  |  |  |  |  |  |  |  |  |
| Muslim | 1.66 | | 0.005 | 1.17-2.37 |  | 0.95 | 0.018 | 0.90-0.99 |  |  | …. | ….. | …... |  | …. | ….. | …... |
| Christian | 1.17 | | 0.13 | 0.96-1.43 |  | 0.98 | 0.190 | 0.95-1.01 |  |  |  |  |  |  |  |  |  |
| **Sex: Age group (years)** |  | |  |  |  |  |  |  |  |  |  |  |  |  |  |  |  |
| Male*15-19 | 1 | |  |  |  |  |  |  |  |  | 1 |  |  |  |  |  |  |
| Female*20-24 | 0.64 | | <.001 | 0.52-0.80 |  |  |  |  |  |  | 0.62 | <.001 | 0.50-0.77 |  |  |  |  |
| **Sex: Level of formal education** |  | |  |  |  |  |  |  |  |  |  |  |  |  |  |  |  |
| Male*No education | 1 | |  |  |  |  |  |  |  |  |  |  |  |  |  |  |  |
| Female*Primary education (1-4/5-7) | 0.77 | | 0.043 | 0.60-0.99 |  |  |  |  |  |  |  |  |  |  |  |  |  |
| Female* Secondary or higher education | 0.83 | | 0.244 | 0.60-1.14 |  |  |  |  |  |  |  |  |  |  |  |  |  |
| **Sex: Marital status** |  | |  |  |  |  |  |  |  |  |  |  |  |  |  |  |  |
| Male*Never married |  | |  |  |  | 1 |  |  |  |  |  |  |  |  |  |  |  |
| Female*Monogamous/ polygamous |  | |  |  |  | 0.99 | 0.563 | 0.95-1.03 |  |  |  |  |  |  |  |  |  |
| Female*Widow/Separated |  | |  |  |  | 0.85 | 0.038 | 0.73-0.99 |  |  |  |  |  |  |  |  |  |
| **Sex: Employment status** |  | |  |  |  |  |  |  |  |  |  |  |  |  |  |  |  |
| Male*Unemployed | 1 | |  |  |  | 1 |  |  |  |  | 1 |  |  |  |  |  |  |
| Female*Student | 0.30 | | <.001 | 0.16-0.55 |  | 1.12 | 0.002 | 1.04-1.20 |  |  | 0.38 | 0.002 | 0.21-0.69 |  |  |  |  |
| Female*Employed | 0.46 | | 0.010 | 0.25-0.83 |  | 1.10 | 0.006 | 1.03-1.19 |  |  | 0.61 | 0.098 | 0.34-1.10 |  |  |  |  |
| **Surv 7 [2012/2013]** | | | | | | | | | | | | | | | | | |
| **Age group (years)** |  | |  |  |  |  |  |  |  |  |  |  |  |  |  |  |  |
| 15-19 | 1 | |  |  |  | …. | ….. | …... |  |  | 1 |  |  |  |  |  |  |
| 20-24 | 0.85 | | 0.101 | 0.71-1.03 |  |  |  |  |  |  | 1.76 | <.001 | 1.47-2.11 |  | 1.17 | <.001 | 1.15-1.19 |
| **Sex** |  | |  |  |  |  |  |  |  |  |  |  |  |  |  |  |  |
| Male | 1 | |  |  |  | 1 |  |  |  |  | 1 |  |  |  |  |  |  |
| Female | 2.24 | | <.001 | 1.49-3.37 |  | 0.89 | <.001 | 0.84-0.94 |  |  | 1.35 | 0.097 | 0.95-1.92 |  | 0.96 | <.001 | 0.94-0.97 |
| **Residence** |  | |  |  |  |  |  |  |  |  |  |  |  |  |  |  |  |
| Rural | …. | | ….. | …... |  | …. | ….. | …... |  |  | …. | ….. | …... |  | …. | ….. | …... |
| peri-urban/urban |  | |  |  |  |  |  |  |  |  |  |  |  |  |  |  |  |
| **Marital status** |  | |  |  |  |  |  |  |  |  |  |  |  |  |  |  |  |
| Never married | 1 | |  |  |  | 1 |  |  |  |  | 1 |  |  |  |  |  |  |
| Monogamous/ polygamous | 0.85 | | 0.232 | 0.66-1.11 |  | 1.06 | 0.003 | 1.02-1.11 |  |  | 1.02 | 0.874 | 0.79-1.31 |  | 0.99 | 0.842 | 0.91-1.08 |
| Widow/Separated | 0.67 | | 0.498 | 0.21-2.12 |  | 0.98 | 0.892 | 0.76-1.26 |  |  | 1.25 | 0.697 | 0.40-3.92 |  | 1.02 | 0.650 | 0.93-1.13 |
| **Level of formal education** |  | |  |  |  |  |  |  |  |  |  |  |  |  |  |  |  |
| No education | 1 | |  |  |  | 1 |  |  |  |  | 1 |  |  |  |  |  |  |
| Primary education (1-4/5-7) | 1.12 | | 0.265 | 0.92-1.37 |  | 0.97 | 0.031 | 0.94-0.99 |  |  | 0.97 | 0.603 | 0.84-1.10 |  | 0.98 | <.001 | 1.15-1.19 |
| Secondary or higher education | 0.87 | | 0.209 | 0.69-1.08 |  | 1.02 | 0.173 | 0.99-1.05 |  |  | 1.12 | 0.158 | 0.96-1.31 |  | 1.02 | 0.046 | 1.00-1.04 |
| **Employment status** |  | |  |  |  |  |  |  |  |  |  |  |  |  |  |  |  |
| Unemployed | 1 | |  |  |  | 1 |  |  |  |  | 1 |  |  |  |  |  |  |
| Student | 0.83 | | 0.279 | 0.60-1.16 |  | 1.00 | 0.839 | 0.96-1.05 |  |  | 0.62 | 0.003 | 0.44-0.85 |  | 1.07 | 0.026 | 1.01-1.13 |
| Employed | 1.35 | | 0.056 | 0.99-1.83 |  | 0.97 | 0.124 | 0.93-1.01 |  |  | 1.13 | 0.423 | 0.83-1.54 |  | 0.90 | <.001 | 0.86-0.94 |
| **Religion** |  | |  |  |  |  |  |  |  |  |  |  |  |  |  |  |  |
| Traditional/other | 1 | |  |  |  |  |  |  |  |  |  |  |  |  |  |  |  |
| Muslim | 1.15 | | 0.509 | 0.77-1.71 |  | …. | ….. | …... |  |  | …. | ….. | …... |  | 1.03 | 0.047 | 1.00-1.06 |
| Christian | 1.29 | | 0.006 | 1.08-1.54 |  |  |  |  |  |  |  |  |  |  | 0.95 | 0.501 | 0.82-1.10 |
| **Sex: Age group (years)** |  | |  |  |  |  |  |  |  |  |  |  |  |  |  |  |  |
| Male*15-19 | 1 | |  |  |  |  |  |  |  |  | 1 |  |  |  |  |  |  |
| Female*20-24 | 0.74 | | 0.014 | 0.58-0.94 |  |  |  |  |  |  | 0.68 | 0.002 | 0.53-0.86 |  |  |  |  |
| **Sex: Marital status** |  | |  |  |  |  |  |  |  |  |  |  |  |  |  |  |  |
| Male*Never married | 1 | |  |  |  | 1 |  |  |  |  | 1 |  |  |  |  |  |  |
| Female*Monogamous/ polygamous | 2.16 | | <.001 | 1.58-2.97 |  | 0.89 | <.001 | 0.85-0.94 |  |  | 1.44 | 0.020 | 1.06-1.96 |  |  |  |  |
| Female*Widow/Separated | 2.37 | | 0.159 | 0.71-7.94 |  | 0.98 | 0.885 | 0.76-1.27 |  |  | 1.14 | 0.832 | 0.34-3.76 |  |  |  |  |
| **Sex: Level of formal education** |  | |  |  |  |  |  |  |  |  |  |  |  |  |  |  |  |
| Male*No education | 1 | |  |  |  | 1 |  |  |  |  |  |  |  |  |  |  |  |
| Female*Primary education (1-4/5-7) | 0.68 | | 0.005 | 0.51-0.89 |  | 1.09 | <.001 | 1.05-1.14 |  |  |  |  |  |  |  |  |  |
| Female* Secondary or higher education | 0.84 | | 0.287 | 0.61-1.16 |  | 1.07 | 0.005 | 1.02-1.11 |  |  |  |  |  |  |  |  |  |
| **Sex: Employment status** |  | |  |  |  |  |  |  |  |  |  |  |  |  |  |  |  |
| Male*Unemployed | 1 | |  |  |  | 1 |  |  |  |  | 1 |  |  |  |  |  |  |
| Female*Student | 0.32 | | <.001 | 0.21-0.49 |  | 1.12 | <.001 | 1.06-1.18 |  |  | 0.41 | <.001 | 0.27-0.62 |  |  |  |  |
| Female*Employed | 0.70 | | 0.049 | 0.50-1.00 |  | 1.05 | 0.050 | 1.00-1.11 |  |  | 0.89 | 0.517 | 0.63-1.26 |  |  |  |  |
| **Surv 8 [2015/2016]** | | | | | | | | | | | | | | | | | |
| **Age group (years)** |  | |  |  |  |  |  |  |  |  |  |  |  |  |  |  |  |
| 15-19 | 1 | |  |  |  | …. | ….. | …... |  |  | 1 |  |  |  |  |  |  |
| 20-24 | 0.84 | | 0.091 | 0.69-1.03 |  |  |  |  |  |  | 1.71 | <.001 | 1.41-2.09 |  | 1.16 | <.001 | 1.14-1.18 |
| **Sex** |  | |  |  |  |  |  |  |  |  |  |  |  |  |  |  |  |
| Male | 1 | |  |  |  | 1 |  |  |  |  |  |  |  |  |  |  |  |
| Female | 3.28 | | 0.001 | 1.66-6.49 |  | 0.87 | 0.002 | 0.79-0.95 |  |  | 2.31 | 0.016 | 1.17-4.57 |  | 0.97 | <.001 | 0.96-0.98 |
| **Residence** |  | |  |  |  |  |  |  |  |  |  |  |  |  |  |  |  |
| Rural | …. | | ….. | …... |  | …. | ….. | …... |  |  | …. | ….. | …... |  | …. | ….. | …... |
| peri-urban/urban |  | |  |  |  |  |  |  |  |  |  |  |  |  |  |  |  |
| **Marital status** |  | |  |  |  |  |  |  |  |  |  |  |  |  |  |  |  |
| Never married | 1 | |  |  |  | 1 |  |  |  |  | 1 |  |  |  |  |  |  |
| Monogamous/ polygamous | 1.01 | | 0.939 | 0.79-1.30 |  | 1.03 | 0.088 | 1.00-1.07 |  |  | 1.24 | 0.002 | 1.08-1.42 |  | 0.98 | 0.635 | 0.91-1.06 |
| Widow/Separated | 1.34 | | 0.477 | 0.60-3.03 |  | 1.01 | 0.881 | 0.90-1.12 |  |  | 1.21 | 0.199 | 0.90-1.63 |  | 1.00 | 0.913 | 0.97-1.03 |
| **Level of formal education** |  | |  |  |  |  |  |  |  |  |  |  |  |  |  |  |  |
| No education | 1 | |  |  |  | 1 |  |  |  |  | 1 |  |  |  |  |  |  |
| Primary education (1-4/5-7) | 1.20 | | 0.045 | 1.00-1.43 |  | 0.98 | 0.448 | 0.94-1.03 |  |  | 1.06 | 0.507 | 0.89-1.27 |  | 0.98 | 0.016 | 0.97-0.997 |
| Secondary or higher education | 1.08 | | 0.460 | 0.89-1.30 |  | 0.99 | 0.561 | 0.95-1.03 |  |  | 1.16 | 0.136 | 0.95-1.40 |  | 1.02 | 0.003 | 1.01-1.04 |
| **Employment status** |  | |  |  |  |  |  |  |  |  |  |  |  |  |  |  |  |
| Unemployed | 1 | |  |  |  | 1 |  |  |  |  | 1 |  |  |  |  |  |  |
| Student | 1.06 | | 0.861 | 0.54-2.09 |  | 0.97 | 0.467 | 0.90-1.05 |  |  | 0.73 | 0.371 | 0.37-1.44 |  | 1.01 | 0.583 | 0.99-1.02 |
| Employed | 2.47 | | 0.008 | 1.27-4.81 |  | 0.89 | 0.004 | 0.83-0.96 |  |  | 1.80 | 0.081 | 0.93-3.50 |  | 1.00 | 0.921 | 0.99-1.02 |
| **Religion** |  | |  |  |  |  |  |  |  |  |  |  |  |  |  |  |  |
| Traditional/other |  | |  |  |  |  |  |  |  |  |  |  |  |  |  |  |  |
| Muslim | …. | | ….. | …... |  | …. | ….. | …... |  |  | …. | ….. | …... |  | …. | ….. | …... |
| Christian |  | |  |  |  |  |  |  |  |  |  |  |  |  |  |  |  |
| **Sex: Age group (years)** |  | |  |  |  |  |  |  |  |  |  |  |  |  |  |  |  |
| Male*15-19 | 1 | |  |  |  |  |  |  |  |  | 1 |  |  |  |  |  |  |
| Female*20-24 | 0.68 | | 0.003 | 0.53-0.87 |  |  |  |  |  |  | 0.72 | 0.010 | 0.56-0.93 |  |  |  |  |
| **Sex: Marital status** |  | |  |  |  |  |  |  |  |  |  |  |  |  |  |  |  |
| Male*Never married | 1 | |  |  |  | 1 |  |  |  |  |  |  |  |  |  |  |  |
| Female*Monogamous/ polygamous | 1.5 | | 0.008 | 1.11-2.03 |  | 0.96 | 0.034 | 0.92-0.99 |  |  |  |  |  |  |  |  |  |
| Female*Widow/Separated | 0.91 | | 0.831 | 0.38-2.18 |  | 1.00 | 0.999 | 0.89-1.12 |  |  |  |  |  |  |  |  |  |
| **Sex: Employment status** |  | |  |  |  |  |  |  |  |  |  |  |  |  |  |  |  |
| Male*Unemployed | 1 | |  |  |  | 1 |  |  |  |  | 1 |  |  |  |  |  |  |
| Female*Student | 0.20 | | <.001 | 0.10-0.42 |  | 1.15 | 0.002 | 1.05-1.25 |  |  | 0.26 | <.001 | 0.12-0.53 |  |  |  |  |
| Female*Employed | 0.42 | | 0.013 | 0.21-0.83 |  | 1.12 | 0.006 | 1.03-1.21 |  |  | 0.56 | 0.097 | 0.28-1.11 |  |  |  |  |

HR=hazard ratio; AFT=Accelerated Failure Time; TR=time ratio; RMST=Restricted Mean Survival Time; R. RMST=ratio in restricted mean survival time
